# Glycolysis, tumor metabolism, cancer growth and dissemination. A new pH-based etiopathogenic perspective and therapeutic approach to an old cancer question

**DOI:** 10.18632/oncoscience.109

**Published:** 2014-12-18

**Authors:** Khalid O. Alfarouk, Daniel Verduzco, Cyril Rauch, Abdel Khalig Muddathir, H. H Bashir Adil, Gamal O. Elhassan, Muntaser E. Ibrahim, Julian David Polo Orozco, Rosa Angela Cardone, Stephan J. Reshkin, Salvador Harguindey

**Affiliations:** ^1^ University of Khartoum, Khartoum, Sudan; ^2^ H. Lee Moffitt Cancer Center, Tampa, FL, USA; ^3^ University of Nottingham, Sutton Bonington, Leicestershire, Nottingham, UK; ^4^ Unizah Pharmacy Collage, Qassim University, Unizah, AL-Qassim, King of Saudi Arabia; ^5^ Omdurman Islamic University, Omdurman, Sudan; ^6^ Institute of Clinical Biology and Metabolism, Vitoria, Spain; ^7^ University of Bari, Bari, Italy

**Keywords:** Tumor glycolysis, pH and glycolysis, pH and cancer, proton transport inhibitors, cancer growth, metastatic process, cancer treatment, new paradigm in oncology

## Abstract

Cancer cells acquire an unusual glycolytic behavior relative, to a large extent, to their intracellular alkaline pH (pH_i_). This effect is part of the metabolic alterations found in most, if not all, cancer cells to deal with unfavorable conditions, mainly hypoxia and low nutrient supply, in order to preserve its evolutionary trajectory with the production of lactate after ten steps of glycolysis. Thus, cancer cells reprogram their cellular metabolism in a way that gives them their evolutionary and thermodynamic advantage. Tumors exist within a highly heterogeneous microenvironment and cancer cells survive within any of the different habitats that lie within tumors thanks to the overexpression of different membrane-bound proton transporters. This creates a highly abnormal and selective proton reversal in cancer cells and tissues that is involved in local cancer growth and in the metastatic process. Because of this environmental heterogeneity, cancer cells within one part of the tumor may have a different genotype and phenotype than within another part. This phenomenon has frustrated the potential of single-target therapy of this type of reductionist therapeutic approach over the last decades. Here, we present a detailed biochemical framework on every step of tumor glycolysis and then proposea new paradigm and therapeutic strategy based upon the dynamics of the hydrogen ion in cancer cells and tissues in order to overcome the old paradigm of one enzyme-one target approach to cancer treatment. Finally, a new and integral explanation of the Warburg effect is advanced.

## INTRODUCTION AND PERTINENT HISTORY

In the early 1920s, Otto Warburg observed that cancer cells were highly fermentative. He hypothesized that it was due to a metabolic injury [1,2]. Since the discovery that cancer cells produced large quantities of lactic acid and that extracellular/intratumoral acidification has recently been shown to be a major and fundamental factor in local growth and in the metastatic process, NaHCO_3_ and other alkalinizing agents have been proposed for the treatment of cancer almost a century later [3]. Later on, while at Roswell Park Memorial Institute (RPMI), Carl and Gerty Cori continued the work on *in vivo* carbohydrates in cancer [4]. Further along the same line, during the late 70's and early 80's, and also at RMPI, we continued studying the dynamics of *in vivo* glycolysis and tumor secretion of lactic and pyruvic acids in rats with transplanted tumors as well as the effects of systemic acidification in dogs as an antyglycolitic therapeutic measure and on tumor regressions in mice [5,6].

More recently, PET technology has resuscitated the interest of the scientific community on Warburg initial findings up to the point that a few years ago a new International Society to study tumor metabolism and its anticancer therapeutic possibilities was created, the International Society of Proton Dynamics of Cancer (ISPDC), that has recently evolved to The International Society of Cancer Metabolism (ISCaM) (www.ispdc.eu). In the same line, although tumors have a unique metabolic system and a concerted strategy to survive, grow and metastasize, a phenomenon we have called *“the neostrategy of cancer cells and tissues”* [7,8], the glycolytic metabolism of cancer was under-appreciated for almost a century until a recent rebirth of the fundamental role of tumor microenvironment and glycolysis in cancer growth and progression [9-12]. This has led the scientific community to adopt the differential tumor metabolism as an additional hallmark of cancer [13]. This review and integrated new perspective will first consider a detailed study of every single step of glycolysis, mainly in the cancer context, followed by a unitarian approach to the pathogenesis of glycolysis and pH-related cancer growth and metastasis and a proposal for a new integrated approach to the treatment of malignancy.

### Classical view of metabolism as either anabolic or catabolic

In this regard, glycolysis is the cytoplasmic utilization of glucose, which is an example of a catabolic pathway. Normally, glycolysis finishes with the entrance of pyruvate into the Krebs cycle and the mitochondrion in the presence of oxygen. Under certain circumstances, such as an insufficient supply of oxygen, pyruvate is converted to lactate and pumped out of the cell. In cancer cells, the conversion of pyruvate into lactate takes place even in the presence of oxygen (aerobic glycolysis), and this was called the ‘Warburg Effect’ after it was so termed by Racker [14] and has also been known through the years as “the first law of cancer biochemistry” [15]. Warburg defended all his life that the aerobic glycolysis of tumors was “the primary cause of cancer”. However, time has proven this not to be true [8,16]. Among the many proposed mechanisms to explain the metabolic transformation resulting in the Warburg Effect include: (i) adaptation to transient hypoxia, (ii) insulin resistance [9,17], (iii) abnormal enzyme content, (iv) abnormal enzyme activity or isoenzymatic alterations, (v) problems of compartmental transport translocation of pyruvate to the mithocondria), (vi) abnormal content in the number or quality of mitochondria, (vii) abnormal electron transport and ATP production, and (viii) oncogenes and suppressor genes [18]. Recently, intracellular alkalinity have been gaining increasing importance as a simple and integral approach to explain the Warburg phenomenon [8,19].

In this review we will first outline in detail the different steps of glycolysis and then interrelate them with cancer growth and progression.

### Glycolysis

Glycolysis is the metabolic pathway that converts glucose, C_6_H_12_O_6_, into pyruvate, lactate and hydrogen ions (protons). The free energy released in this process is used to form the high-energy compounds, ATP (adenosine triphosphate) and NADH (reduced nicotinamide adenine dinucleotide). Glycolysis takes place in the cytoplasm. It can be directly represented by the following equation [20]:

C_6_H_12_O_6_+ 2NAD^+^ + 2ADPMg^−^ + 2HxPO_4_^3–x−^ → 2CH3CCOCO_2_^−^+ 2 NADH + 2 ATPMg^2−^ + 2 H_2_O + 2xH^+^.

Many allosteric factors of the most varied natures (hormonal, ionic, viral physical, chemical, genetic and metabolic) have been described that regulate glycolysis and/or glucose consumption (Table 1). For more than 30 years, mainly from the sixties to the nineties, an “epidemic of allosteria” predominated in glycolysis research in the many attempts to find an explanation to Warburg's aerobic glycolysis [21].

**Table 1:** Allosteric factors regulating glucose consumption by normal and/or cancer cells

Hormonal	insulin, adrenal steroids, epinephrine, androgens, estrogens, parathyroid hormone, human growth hormone, glucagon, melatonin
Ionic	P_i_, Mg^++^, K^+^, Ca^++^, H^+^.
Viral	reovirus, Rous sarcoma virus, Human papilloma virus E16
Physical	O2, temperature
Chemical	Iodoacetic acid, sacaric acid, sodium folluoride, NH_4_^+^, HIF-1
Genetic	Chromosome 21
Metabolic	ATP, ADP, AMP, citrate, Krebs cycle intermediates, Ketone bodies, Thiamine, Fatty acids, 2-4-dinitrophenol, glucose-5-phosphate, fructose 1-6-biphosphate, bioflavonoids, dietetic sugars, folic acid, phosphocreatine, 3-phosphoglycerate phosphoenolpyruvate, fructosebiphosphatase, 3-5 cyclic AMP, methylglyoxal
Oncogenes	tumor supressor genes
Therapeutic drugs	Methotrexate, clotrimazole

***Modified from Harguindey S. [317]

The whole process of glycolysis takes place through two phases: a preparatory phase and a harvesting phase.

### 1. Investment, preparatory phase

#### 1.1 First step. Glucose fixation

**Figure 1:**
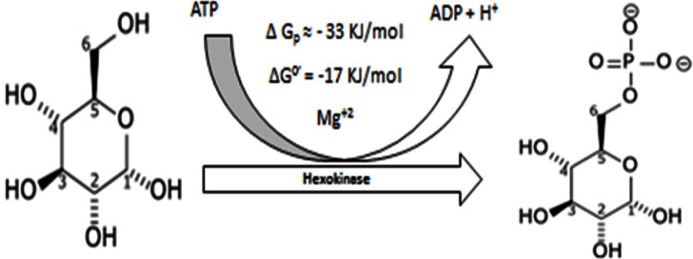
Conversion of Glucose into Glucose – 6 – Phosphate

In this step, glucose is fixed intracellularly by the addition of a phosphate group to form glucose-6-phosphate and such phosphorylation prevents glucose efflux. This requires the addition of a phosphate group from ATP and needs hexokinase or glucokinase as a catalyst for this reaction. This step requires Mg^+2^ as co-factor. It requires about −17 KJ/mole under normal conditions [22] (Figure 1) (where ∆G^0^ is the free energy of a reaction and the minus (−) sign shows this reaction to be exothermic i.e. releases energy) (Figure 1). It is most likely that hexokinase has an anti-apoptotic function, which is why it might explain its overexpression in tumors [23]. Precisely, this enzyme could be a glucokinase rather than hexokinase because it is not inhibited allosterically [24].

Therapeutic targeting of this step:

1. Glucose transport inhibitors: glucose is taken up by the glucose transporters Glut-1 or the sodium-dependent glucose transporters e.g. sodium-dependent glucose cotransporter-1 or sodium-glucose linked transporter 1 (SGLT1). Glut-1 and SGLT-1 are overexpressed in cancer [25]. Targeting the Glut-1 transporter [26] represents a potential anti-tumor strategy directed towards glucose deprivation [27]. Also, it sensitizes cancer cells to death receptors by arresting them in G_0_-G_1_ phase [28], so re-sensitizing them to a death ligand like TRAIL.
2. At the substrate level, therapeutic attempts have been tried by administering “fake” substrates which are not normally reversed by insulin activity; e.g. 2-Deoxy-D-glucose (2-DG) [29-31]. Glucosamine also inhibits hexokinase [32]. Furthermore, metrizamide also inhibits hexokinase [33] but to a lesser degree in comparison to 2-DG and glucosamine [32].
3. Chromosomal amplification also is another strategy to stimulate glycolysis [34] which could be targeted through siRNA [35].

In summary, targeting hexokinase represents a seductive strategy in treating cancer, e.g. with Mannoheptulose [36]. However, another main concern in solid tumors is the problem of those enzymatic inhibitors reaching all tumor areas as they probably reach only the tumors outer layers because of low O_2_ tension and low blood supply, so impairing drug diffusion [37]. Although the following step is not reversible, the glucose-6-phosphatase enzyme exports glucose extracellularly [38]. Vanadium is also a potential inhibitor of the phosphatase enzyme [39]. However, targeting this enzyme remains of doubtful value due to its variable expression in tumors [40,41].

#### 1.2 Second step: Gluco-Fructose isomerization

**Figure 2:**
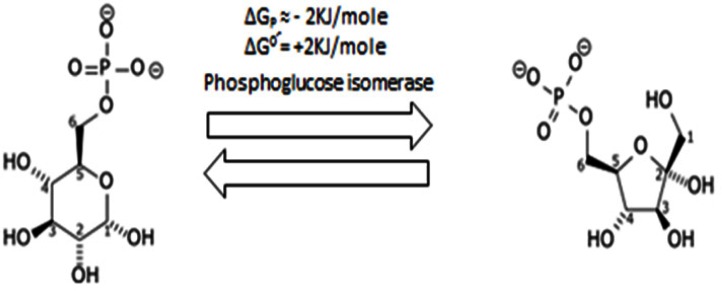
Shows isomerization of Glucose-6-phosphate into fructose-6-phosphate

In this step Anti-AMF (Autocrine Motility factor) antibodies are correlated with arthritis and considered arthritogenic [42-44]. Its role in chronic inflammation might collaborate in inflammation-related carcinogenicity [45,46] since it is known that glucose-6-phosphate is isomerized (intramolecular reaction, rearrangement) into fructose-6-phosphate by Glucose-6-phosphate isomerase (phosphoglucose isomerase or phosphohexose isomerase) (Figure 2). This is a reversible reaction following Le Chatelier's principle. This principle determines that in a reversible reaction, when the concentration of a reactant/s exceeds the concentration of the product/s, the reaction goes in the direction that produce more product/s and decrease the concentration of the reactant/s (it goes to the right). Parallel to the context, if the concentration of the product/s exceeds the concentration of the reactant/s, the reaction goes in the reverse direction to produce more reactant/s (it goes to the left). This is, when found extracellularly, Glucose-6-phosphate isomerase is a synonym of (I) Neuroleukin (neurotrophic factor) [47], and (II) AMF [48]. Therefore, Glucose-6-phosphate isomerase is an example of an ectoenzyme (exoenzyme) [49]. Early studies using chromatography suggested that AMF has two active peaks at different pH values [50] that might be compatible with the uniqueness of the cancer cell reversed pH gradient [51], a selective hallmark of they having an alkaline cytosol and an acidic extracellular microenvironment [37,51]. Therefore, AMF facilitates glycolysis inside the cells as a downhill reaction to produce lactate and H+, which facilitates its role outside the cells as a tool for invasiveness; *i.e.* AMF has a dual role, first by activating glycolysis and then by collaborating in inducing an extracellular acidity that promotes tumor invasiveness and metastasis [11,52,53]. So, AMF plays a critical role in neoplastic transformation [54], invasiveness [55,56], and metastasis [57,58].

#### 1.3 Third step. Commitment to glycolysis

**Figure 3:**
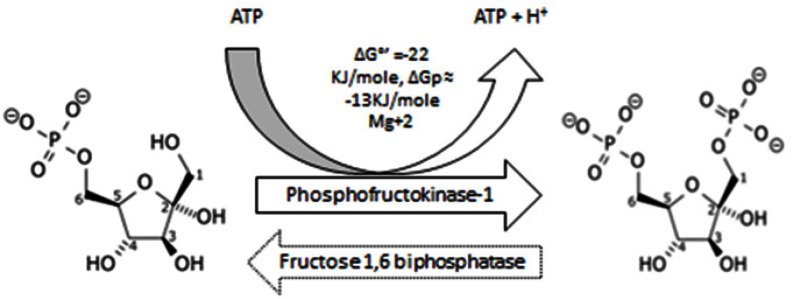
Shows phosphorylation of Fructose – 6-Phosphate into Fructose – 1, 6 – biphosphate

Fructose-6-phosphate is further phosphorylated into unstable molecules termed “fructose-1,6-biphosphate” (Figure 3), and fructose-2,6-biphosphate (relatively stable than the other ones due to less steric hindrance) respectively by phosphofructokinase-1 (PFK-1) and phosphofructokinase-2 (PFK-2).

##### Fructose-1, 6-Biphosphate

Fructose-1,6-biphosphate, also known as Harden-Young ester, is formed during glycolysis by phosphofructokinase-1 (PFK-1) and requires Mg^+2^ and an ATP molecule.

Fructose-1,6-biphosphate activates pyruvate kinase allosterically [59,60]. Fructose-1,6-biphosphate has a cytoprotective activity by chelation of iron [61] and also behaves as a free radical scavenger [62-65]. In this regard, fructose-1,6-biphosphate may play a critical role in the prevention of programmed cell death (apoptosis). Therefore, it is possible that this glycolytic intermediate could be involved in certain cases of *de novo* multiple drug resistance (MDR) and be functionally equivalent to glutathione in that “its absence might promote carcinogenesis but its elevation can confer chemotherapy resistance” [66]. Also, both fructose-1,6-biphosphate and glutathione are interlinked together as a survival strategy against hypoxia [67]. Furthermore, fructose-1,6-biphosphate inhibits T-cell proliferation and has anti-inflammatory properties through inhibition of interleukin 1,6 and beta-catenin [68]. Therefore, on one hand, it inhibits immune response, which supports tumors fitness, while on the other hand it attenuates the inflammatory environment which generally alters tumor progression [69]. All in all, its precise role remains mostly undefined.

Glucose-6-phosphate isomerase is an inflammation-inducing agent while fructose-1,6-biphosphate is a free radical scavenger and an anti-inflammatory enzyme.

##### Phosphofructokinase-1 (PFK-1)

PFK-1 is a tetramer enzyme that consists of three subunit types: muscle (M or A), liver (L or B) and platelets (P or C) [70,71]. Phosphofructokinase of muscle is composed of homotetramer 4M, the liver predominantly contains the L subunit in addition to M and P subunits. Brain and heart exhibit three subunits [72,73], which correlates with differences in tissue specialization. It has been shown that tumors over-express PFK-1 and preferentially its L-subunit [74]. PFK-1 might be useful for monitoring of progression of some cancers and also to identify tumor stage [75].

In 1986, it was demonstrated in some strains of rats that they only have M subunits in their muscles but that other organs differ drastically in tissue/organ proportion in PFK-1 subunit expression [72]. From this study a question was raised concerning if there is any possibility that human ethnicity could lead to differences in tissue/proportion of PFK-1. If this diversity is present it could affect tumor behavior in certain cases. This could help to study tumor progression in terms of ethnicity and management of cancer is different human populations.

##### PFK-1 regulation

Interestingly, PFK-1 has the same kinetic characteristics in both aerobic and anaerobic conditions [76]. Also, a slightly alkaline pH_i_ is the optimum to maximize PFK-1 activity [52,77-80]. On one hand, it has been known for decades that an alkaline pH_i_ even slightly above steady-state levels stimulates the activity of this key glycolytic enzyme and inhibits gluconeogenesis. Indeed, in cancer cells a high pH_i_ situation can increase the allosteric regulation of PFK-1 more than a 100-fold and even a raise of 0.2 pH units can convert this enzyme from an inactive form to a fully active quaternary structure [5,80-83]. This post-Warburg, H^+^-related approach to glycolysis and tumour metabolism has originated during the last few years a completely and integral new paradigm in approaching oncological metabolic research and cancer treatment based upon the hydrogen ion dynamics of cancer cells and tissues [10,11,52,53,84-89]

Furthermore, phosphocreatine inhibits PFK-1 [90] while 3-phosphoglycerate and phosphoenolpyruvate act synergistically with ATP to inhibit PFK-1 [90,91]. Finally, ADP, among other factors, activates PFK-1 allosterically [92,93] (see Table 1). PFK-2 also activates PFK-1 through fructose 1,6 biphosphate, but during persistent exercise in frog muscle, fructose-2,6 biphosphate levels drop, while P_i_, AMP and ADP all activate PFK-1 during exercise [94]. Thus, in persistent exercise, normal cell physiology relies on endogenous activators in order to maintain energetic requirements and not only on fructose 2,6-biphosphate activity. Finally, clotrimazole has anti-PFK-1 activity *in vitro* [95].

##### Fructose 1,6-biphosphatase (FBPase-1)

Fructose-1,6-biphosphatase is one of the key enzymes that mediates gluconeogenesis. It has two isoforms: Liver (L-FBPase) and muscle (M-FBPase) [96-100]. Insulin decreases expression of FBPase-1 [101]. Although cAMP increases expression of FBPase-1 [101], AMP strongly inhibits FBPase-1 [102,103]. Fructose-1,6–biphosphate inhibits FBPase-1 [104] by acting synergistically with Fructose-2,6–biphosphate [105]. Also, Fructose-2,6–biphosphate acts synergistically with AMP to inhibit FBPase-1, and this inhibition is decreased at higher substrate concentrations [105]. The same study pointed out that alkaline pH decreases the inhibitory effect of Fructose-2,6 –biphosphate [105] while FBPase-1 activity is increased at higher, alkaline pH_i_ [106].

##### Role of FBPase-1 in mediating resistance

It has been shown that phosphofructokinase is down-regulated while FBPase-1 is up-regulated in radiation resistant cell lines. Both features together suppress apoptosis through increasing glutathione levels [107]. In this context, the PFK-1/PFBase ratio plays a critical role in tumor proliferation and/or tumor resistance ratio while FBPase over-expression could be considered to be one important adaptive strategy of resistance. In other words, by providing more lactate, glycolysis supplies an evolutionary advantage [108] as well as a metabolic resource [9]. However, over-expression of gluconeogenic enzymes during resistance might support the decrease in glycolysis and so a reduction in proliferation rates, which is an adaptative cost of resistance. Expression of FBPase-1 leads to the formation of glucose-6-phosphate and therefore feeds the pentose phosphate pathway (PPP) [107,109].

##### Fructose - 2, 6-biphosphate

Studying the mechanism of action of glucagon on gluconeogenesis led to the discovery of Fructose-2,6-biphosphate [110,111]. This molecule is crucial in maintaining the glycolysis downward chain reaction and increasing commitment to glycolysis, especially when ATP levels are raised [112]. This step is irreversible. Since, as described above, PFK-1 is inactive under physiological conditions and is activated by Fructose 2,6 biphosphate synergistically with AMP [113], it seems that Fructose 2,6-biphosphate has evolved in order to enhance insulin activity [114] and so increase glucose uptake. Finally, palmitate decreases the level of Fructose-2, 6-biphosphate [115].

##### PFK-2

When the glucose level is decreased, glucagon is secreted to activate cAMP as a consequence of adenylcyclase activation [116,117]. cAMP activates FBPase-2 [118] and activation of FBPase-2 leads to a decrease of fructose 2,6-biphosphate [119]. In turn, reduction of fructose 2,6-biphosphate leads to inactivation of PFK-1 and, thus, stimulates gluconeogenesis [120]. On the other hand, insulin has a reverse effect. Glucose fixation is increased and also up-regulation of glycolysis as a result of the binding of glucokinase to PFK-2/FBPase-1 [121], which indicates enzymatic interactions.

In cardiac muscle, PFK-2 is a cellular defense strategy against hypoxia [122] and PFK-2 deficiency is correlated to insulin resistance [123]. Also, citrate inhibits PFK-2 to a greater extent than PFK-1 [124]. Therefore, the citrate that results from the Krebs' cycle inhibits PFK-2 [125], which suggests a negative feedback mechanism. Phosphoenolpyruvate also inhibits PFK-2 [126]. Although it is a commitment step, production of energy is a key determinant factor to finish this step. In this way, glycolysis is tightly regulated internally. It has also been demonstrated that protons inhibit PFK-2 [78,127] while an alkaline pH_i_ increases its activity [128].

*PFK-2 has 4 isoenzymes*: PFKFB 1, 2, 3, 4 [129,130]. PFKFB3 and 4 are correlated with cancer and their expression is higher in metastasis as compared to primary tumors [131,132]. Interestingly, Hypoxia Inducible Factor-1 alpha (HIF-1 alpha) increases transcription of PFKFB4 [133]. Altogether, these data suggest that they could potentially become important antimetastatic targets [131]. However, the targeting of PFK-2 should be carefully monitored because it might shift to complete glycolysis through PPP; *i.e.* as a provider of energy with anti-oxidant capacity (energy plus anti-apoptosis).

As PFK-1 is inactive under physiological conditions [113] and it is activated synergistically by Fructose-2,6-biphosphate together with AMP, one could expect that PFK-2 should be activated in the first place followed by PFK-1 activation. There is a great deal of literature describing that the PPP serves cancer cells to produce nucleic bases; *i.e*. via the formation of cellular building blocks. Although this is an acceptable hypothesis, yet we will now follow a different approach to complement the significance of the PPP pathway.

1.PPP is a very critical pathway because it is capable of forming Glutathione-S-Transferase (GST). GST is a Reactive Oxygen Species (ROS) scavenger. ROS are carcinogens that induce DNA-mutations but upon carcinogenesis ROS prevents the induction of apoptosis. Therefore, PPP is essential for tumor cell immortality. Inhibition of Glucose-6-Phospahte Dehydrogenase (G6PD), which is a key determinant step of PPP, results in the prevention or slowing of carcinogenesis [134].

2. PPP completes glycolysis by consuming 1 ATP instead of 2 ATP molecules and, therefore, it is more energy-efficient than glycolysis. Each molecule of glucose uses 2 ATP and produces 4 ATP molecules. So, the net energy is 2 molecules. Pentose Phosphate pathway does consume 1 ATP molecule and does produce 4 ATP molecules. Therefore, the net result is 3 ATP molecules. In this regard, PPP provides more energy, in addition activation of PPP results in the biosynthesis of nucleic acids. So, cancer cells might decide whether to get pyruvate through the complete glycolysis pathway or rely partially on PPP. Until that decision takes place, PFK-2 might also be an endogenous ROS-sensor to determine whether F-1,2-BP is at appropriate levels or decide to finish glycolysis through PPP and so prevent formation of F-1,6-BP.

##### FBPase-2

PFK-2/FBPase-2 is a bifunctional enzyme and FBPase is reciprocal to PFK-2. Insulin inhibits it in both liver and muscle while epinephrine activates it in muscle and inhibits it on liver [135,136]. Therefore, the metabolic and/or oxidative stress status of the cell could play a role in regulating the relative PFK-2/FBPase-2 cellular activity.

These relationships raise the question of why is it that normal cells do not have the isomerase enzyme instead of A kinase and phosphatase, so that they can save one molecule of ATP. In other words, since developing the isomerase enzyme that can translocate a phosphate group is less expensive than developing a set of enzymes that consume additional ATP molecules, why do normal cells acquire kinase/phosphatase enzyme?

Potential answers are as follows:

1. One of the proposed answers is that fructose 2,6-biphosphate acts as cellular regulator as well as a reservoir. Further, bifunctional enzymes are tightly correlated with function and isomerases require a reversible reaction that is not controlled by external signaling. In this context, many basic researchers and clinicans might consider Fructose 2,6-biphosphate as an Achilles heel; however, it is not as simple as that since Fructose 2,6-biphosphate is a component of various essential cycles.
2. Fructose 1,6-biphosphate is an unstable molecule due to steric hindrance (negative charge of phosphate as a bulky group at carbons 1 and 6. This molecule is reversibly fragmented into D-glyceraldehyde-3-phosphate and dihydroxyacetone phosphate in addition to the remaining steps of glycolysis which are reversible too (except the last one). Therefore, shifting from the product into fructose 2,6-biphosphate prevents fructose 1,6-biphosphate accumulation and, consequently pushes glycolysis to move downward instead of reversing to a gluconeogenic direction. This answer is highly compatible with a role of fructose-2,6-biphosphate as a glycolytic directing agent. In essence, fructose 2,6-biphosphate could be considered as as “guardian of glycolysis”. The most striking example is insulin. Insulin increases glucokinase (hexokinase), PFK-2 and Pyruvate Dehdyrogenase (PDH), so facilitating its entrance into the Krebs' cycle. Therefore, insulin promotes the irreversible steps of glycolysis. Insulin also stimulates the Na^+^/H^+^ exchanger isoform 1 (NHE1) and increases pH_i_ [137].

#### 1.4 Fourth step. Fission step

**Figure 4:**
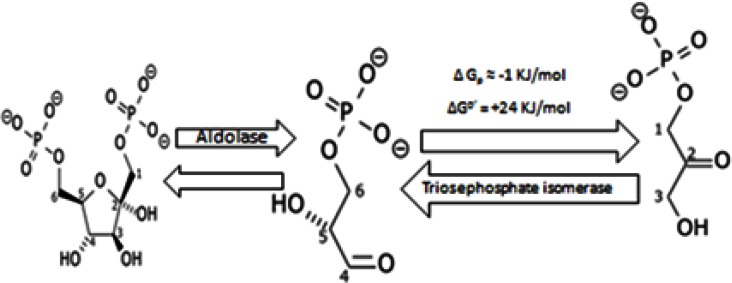
hydrolysis of Fructose – 1, 6 – biphosphate into D-glyceraldehyde 3-phosphate (GADP) and Dihydroxyacetone phosphate (DHAP)

Fructose-1,6-biphosphate is degraded into D-glyceraldehyde 3-phosphate (GADP) and Dihydroxyacetone phosphate (DHAP) by the activity of fructose-biphosphate aldolase (Figure 4). DHAP can be reversed into GADP through activity of triose phosphate isomerase. Again, the presence of a negative charge and the double bond at carbon 2 leads to steric hindrance that isomerizes it into GADP by triose phosphate isomerase. DHAP is an intermediate of several biochemical pathways, including the glycerol phosphate shuttle (the glycerol phosphate shuttle is a mechanism that regenerates NAD^+^ from NADH). Importantly, triose phosphate isomerase is overexpressed in tumors and is correlated with hepatic metastasis [138,139] but its expression is decreased when cancer overexpresses drug resistance proteins; e.g. MDR [140]. Triose phosphate isomerase also shows a higher activity at alkaline pH [141].

Furthermore, in the next step, DHAP is converted into glyceraldehyde-3 phosphate (GADP). GADP is a node for several biochemical pathways including glycolysis, gluconeogenesis, PPP, tryptophan biosynthesis and the glycerol-3 phosphate shunt. GADP inhibits PFK-2 and, in this way, might trigger gluconeogenesis. GADP also inhibits caspase-3 activity (anti-apoptotic effect) [142]. Aldolase (fructose diphosphate aldolase) is the enzyme that converts fructose 1, 6 biphosphate into GADP and DHAP and has 3 isoforms: A, B and C [143,144]. Generally, aldolase C is found in the brain [145], aldolase A is the ubiquitous form and the predominant isozyme in muscle while aldolase B is the predominant isozyme in liver and is also expressed in kidney [146,147]. The expression of most aldolase isoforms fluctuate from one tissue to another and in the same tissue from one time to another and in certain tissues at the moment when the tissue acquires diseases. For example, aldolase B is predominant in the liver of neonates, aldolase A increases in the fetal stage and returns back to aldolase B at adulthood [148]. It was reported early on that aldolase content is increased in cancer [149]. Further, aldolase A becomes dominant in hepatoma while aldolase B decreases in hepatoma and gastric cancer [150]. Once again, intracellular alkalinity is the optimum environmental condition for aldolase activity [151].

Aldolase B is also necessary for fructolysis, which is responsible for the formation of glyceraldehyde-3-phosphate from fructose-1-phosphate. Therefore, Aldolase shows a difference in the ratio of FDP (fructose-1-6-biphosphate) and F1P (fructose 1-phosphate) between liver and spleen [152] which may reflect the following:

1. Tumor behavior differs from one organ to another. So, there is no unifying glycolytic microenvironment.
2. It might also reflect the presence of fructolysis in certain tumors (conversion of fructose 1 phosphate into glyceraldehyde-3–phosphate) as some tumors over-express GLUT-5 [153] as the key transporter of glucose [154]. In this regard, tumor cells uptake fructose and phosphorylate it. Conversely, the source of fructose–1-phosphate could form from PPP too. This raises the question if the Warburg Effect should be reappraised as to whether glycolysis in tumors occurs through the preparatory phase and/or the PPP that meets pH-dependant glycolysis in GADP node or is there a ratio between them.

### 2. Pay-off phase (Harvesting Phase)

#### 2.1 Fifth step. Rearrangement process

**Figure 5:**
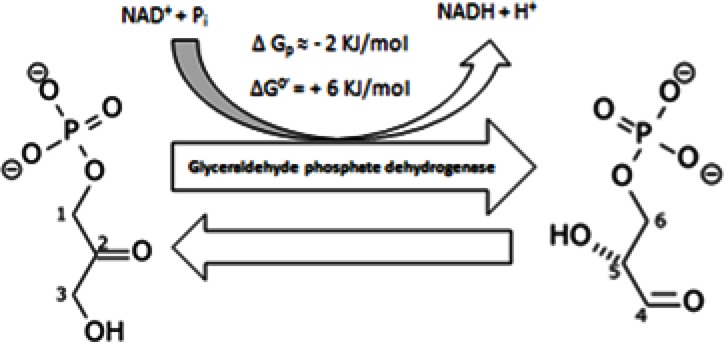
conversion of D-glyceraldehyde 3-phosphate (GADP) into into 1, 3-bisphosphoglycerate

Glyceraldehyde 3-phosphate (GADP) is converted into 1, 3-biphosphoglycerate by the activity of glyceraldehyde phosphate dehydrogenase (GADPH) (Figure 5). This reaction needs NAD^+^ and inorganic phosphate P_i_.

GAPDH has been shown to have apoptotic properties as well as preventing mutagenicity [155]. This raises the question as to what is the evolutionary advantage of its tumor over-expression if it initiates apoptosis? In this respect, HIF1-alpha increases expression of GAPDH [156], raising the question: does HIF1-alpha induce cell death? It is well documented that GAPDH has an apoptotic property, which is why it's over-expression plays a critical role in neurodegenerative diseases and its inhibition serves as a promising strategy for treating such diseases [157-160]. So, the precise question is how does GADPH promote carcinogenesis, as well as inducing neurodegeneration? One possible convincing answer could be that the higher rate of NAD+ production will reduce GADPH capacity by converting it from a tetramer into a dimer [161]. Therefore, glycerol - 3 - phosphate dehydrogenase might play an essential role in producing such an apoptotic reduction. Moreover, PPP reduces ROS that deactivates GADPH [162] and so maintains GADPH levels. In this regard, PPP continues GADPH activity while the glycerol phosphate shuttle attenuates its apoptotic capacity. In the end, tumor cells modulate the dialectic of the contraries of metabolism in an extremely coordinated manner in order to maintain its cellular integrity. Furthermore, it is not sure whether precise modulation comes from lactate dehydrogenase or glycerol–3–phosphate dehydrogenase. In conclusion, Alzheimers Disease and other neurodegenerative diseases have an opposing pathogenesis as compared to cancer, e.g. intracellular acidity might aggravate neurodegenerative capacity as it has been previously suggested [7,163,164]. Finally, GAPDH is inhibited by Monochloroacetate (MCA) [165]. Indeed, intracellular alkalinity is once again the optimum pH for GADPH activity (pH=8.5) [166].

#### 2.2 Sixth step. First Energy Releasing step

**Figure 6:**
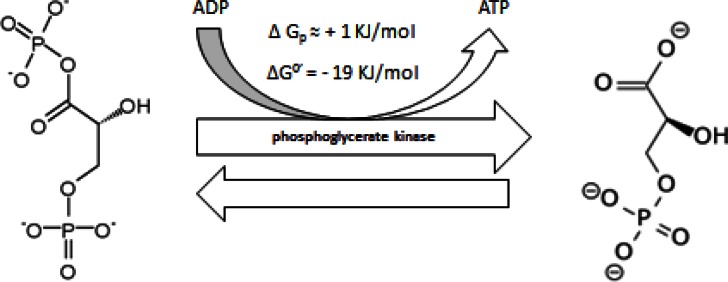
conversion of 1, 3 - bisphosphoglycerate into 3-phosphoglycerate

1,3-bisphosphoglycerate is converted to 3-phosphoglycerate by phosphoglycerate kinase (PGK) and produces ATP at substrate level phosphorylation (Figure 6). PGK has several isozymes [167,168] and its expression rises during anoxia [169] which reflects its preferential expression at anoxic areas of solid tumors [9,108]. However, while some data has shown that PGK-overexpression is correlated with disseminated cancer [170], other data shows an inverse correlation with tumor incidence and consider that PGK enhances an anti-tumor effect because of its anti-inflammatory and anti-angiogenic activity [171].

Regardless of our recent criticism on the anti-angiogenic approach and its possible correlation with a damaging selection of a hypoxic/anoxic phenotype [108], such contradictory evidence could be solved by considering it as a delicate balance between plasminogen activation and inhibition in extracellular matrix (ECM) turnover [172], especially as PGK regulates urokinase receptor expression, which is involved in ECM remodeling, cell proliferation and migration as well as in modulating cell adhesion [173]. Therefore, in targeting PGK the exact determination of the timing of administration of the inhibitory compound is required, depending on whether the goal is to activate or inhibit ECM turnover, quantify oxygenated/hypoxic areas, or manipulate the anoxic ratio of the tumor population.** PGK also shows maximum activity at a cellular pH range similar to that of cancer cells [174,175], which is alkaline [176].

#### 2.3 Seventh Step

**Figure 7:**
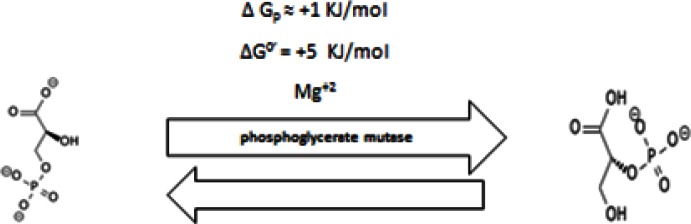
Shows conversion of into 3-phosphoglycerate into 2-phosphoglycerate

3–Phosphoglycerate undergoes structural isomerization and yields 2-phosphoglycerate by phosphoglycerate mutase (PGM) (Figure 7). PGM is overexpressed in cancer and is correlated with poor prognosis [177]. The maximum activity of PGM also occurs at alkaline pHs [178].

#### 2.4 Eight Step

**Figure 8:**
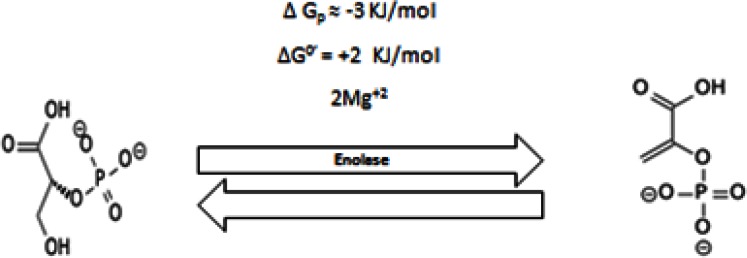
shows conversion of 2-phosphoglycerate into Phosphoenolpyruvate

2–Phosphoglycerate is converted into Phosphoenolpyruvate by the enolase enzyme (Figure 8). In mammalian cells, there are three independent genetic loci: α, β and γ. They code and express three different isozymes according to tissue specificity. Alpha enolase (ENO1) is found in most adult tissues, beta enolase (ENO3) is found in muscle and gamma enolase (ENO2) is found in the brain [179].

It has also been postulated that the enolase has tumor suppressive properties [180] as it is absent in some tumors [181] while other data supported that it is overexpressed in some malignant tumors [182]. One of the possible answers for this inconsistency comes through the understanding of the subcellular localization of enolase and its translational process. Enolase has been found at the cell surface as a plasminogen binding protein, which was found to promote tumor invasiveness and metastasis [183], boosting immunization to prevent bacterial virulence [184-189]. Besides, at the cellular membrane enolase has also been found at the cytoplasm and nucleus [190]. The *ENO-1* gene is responsible for expression of enolase-1 as well as for Myc-binding protein-1 (MBP-1); that is, the same gene provides two different proteins at the translational level [191,192].

Enolase is very crucial in completing glycolysis and, thus, so might promote tumorigenesis while MBP-1 blocks the activity of c-myc expressing protein [193,194]. Therefore, the key determinant that instigates either tumor growth or tumor regression is translation of *ENO-1* gene towards Enolase or MBP-1 expression respectively (Enolase/MBP-1 ratio). Such an evolutionary fate might be determined through microenvironmental selection, e.g. hypoxia. Tumor hypoxia preferentially selects translation of enolase and attenuates that of MBP-1 [195]. Moreover, the presence of hypoxia increases the production of ROS and the expression of c-myc [195]. C-myc increases production of mitochondrial ROS and it has been shown to stabilize HIF1-alpha [196,197] suggesting a delegate balance between C-myc, ROS and HIF1-alpha in maintaining cellular survival and tumor progression. In conclusion, ENO-1 expresses different proteins that can be localized either in the nucleus as a tumor repressor [191] or in the cytosol as a glycolytic enzyme or, finally, at the cell surface, where it promotes invasiveness and metastasis [198]. It has a higher activity at pH 7.5 when in phosphate buffer [199].

#### 2.5 Ninth Step (Formation of Pyruvate)

**Figure 9:**
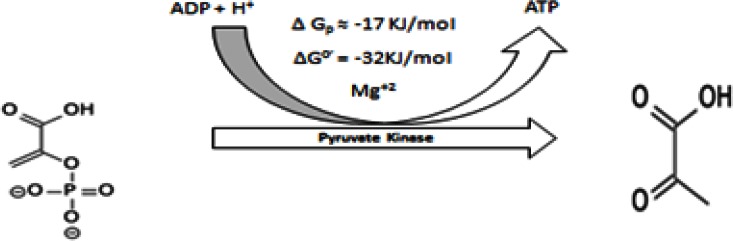
shows conversion of Phosphoenolpyruvate into Pyruvate

Phosphoenolpyruvate (PEP) is converted into pyruvate through pyruvate kinase enzymes (Figure 9). This second step produces ATP at substrate level phosphorylation. Pyruvate kinase has four isozymes PK L, R, M_1_, and M2 (Table 2) [200,201]. L-alanine is a strong inhibitor of PK-L and hepatoma and has little effect on PK-M while phenylalanine inhibits PKM [202]. Phosphoenolpyruvate activates pyruvate kinase and fructose 1,6-biphosphate [60]. Epinephrine and glucagon phosphorylate and deactivate PK-L [203] while insulin dephosphorylates enzymes and activates it [204,205]. The ATP/AMP ratio is very important in determining PK activity.

**Table 2:** Distribution of Pyruvate Kinase isoforms among tissues

Pyruvate Kinase Isoforms	Tissue localization
PKL	RBCs
PKR	Liver
PKM1	Muscle
PKM2	Kidney, brain, heart, thymus, spleen, lung, adipose tissue, testis and ovary

***For further details, see text

Although rabbit PEP binding with PKL is increased with increasing pH from 6 to 8.5 [206], the effect of pH on PK activity is very intricate and is dependent on the concentration of allosteric activators, ATP levels and species variation [200,207,208]. This is why small changes in intracellular pH alters PK activity [209]. At least in yeast, protons facilitate PEP binding but weaken binding with Mg^+2^ and ADP [210].

PKM2 can be translocated into the nucleus and induces cellular proliferation [211] unless it binds with other agents to induce apoptosis [211]. One can therefore ask, what is the role of PKM in cells that are undergoing apoptosis? The most convincing answer is that apoptosis is an active process that needs energy [212-214]. Therefore, further work should be done to differentiate how and when the energy produced could be invested for tumor cell proliferation or exploited to undergo programmed cell death.

Malignant progression is accompanied by a decrease in the PKM1/PKM2 ratio, both being different splicing products of the M-gene (exon 9 for PKM1 and exon 10 for PKM2) [215]. Only PKM2 is active when tissue becomes cancerous [216,217]. PKM2 can be found in two forms, dimer and tetramer [218]. The tetramer form has a high affinity for PEP even below physiological concentrations of PEP [218]. In contrast, the dimer has a low affinity for PEP and is nearly inactive at physiological PEP concentrations, being mainly expressed in tumors [219]. The fact that cancer cells can preferentially express the less active form of PKM2 could be due to various possibilities:

A decrease in the conversion of PEP into pyruvate leads to accumulation of glycolytic intermediates at upstream pathways that facilities and encourages the formation of other building blocks such as nucleic acids.

PKM2 encourages lactate formation rather than the entrance into the Krebs cycle; that is to say that the PKM2/PKM1 ratio is directly proportional to the LDH/PDH (Lactate dehydrogenase/Pyruvate dehydrogenase ratio) [220]. The provision of lactate (i) supports regeneration of another NAD^+^ and (ii) increases tumor acidity. In this way, PKM2 promotes tumor fitness, which becomes another evolutionary advantage [32].

Fructose 1,6-biphosphate encourages re-association of the dimer into a tetramer [221]; *i.e.* fructose 1,6-biphosphate decreases the dimer/tetramer ratio and so facilitates entry into the Krebs' cycle which promotes mitochondrial activity as well as decreases lactate production. However, activation of the Krebs' cycle might not necessarily induce apoptosis. In other words, production of ATP through the oxidative phosphorylation system (OXPHOS), might not indicate that mitochondria have re-activated their role as a cell death machinery. However, activation of the Krebs' cycle produces huge amounts of ATP in comparison to glycolysis and when the ATP level is raised, PFK2 is inhibited leading to inhibition of fructose 1, 6-biphosphate. So, it is a negative feedback mechanism.

In conclusion, the dimer/tetramer ratio acts as an auto-sensor that drives the synthesis of nucleic acid and other components at certain times while at other times cancer cells generate energy from mitochondria. So, it will not be a surprise if this ratio is coordinated and synchronized with the cell cycle. Any alteration in this sensor pathway might lead to catastrophic events in cancer at levels of either the individual cell selection or at group selection (tumor population). Endogenous agents that alter this cycle include: (i) tyrosine kinase, that leads to a release of fructose 1,6-biphosphate [216] and (ii) thyroxin, that might disturb or induce sensor noise [222] because cytosolic thyroxin hormone-binding protein (P58) is a monomer of PK subtype M2v [222]. Interestingly, this last possibility might explain the thermogenic activity of thyroxin and its delicate balance and correlation with adaptation of cancer to hypo/hyperthermia. If so, we should look again at the impact of thyroxin diffusion across or through a tumor colony in the same way as for estrogen diffusion [223].

PKM1 has less impact on *in vivo* proliferation than PKM2 [219]. Therefore, again this ratio will not only support our hypothesis of synchronization with the cell cycle but also this ratio is very important in maintaining tumor population density especially under low nutrient conditions, e.g. improper blood supply that leads to adaptation of hypoxia that might increases c-myc and this later might be correlated with PKM2 expression too [224] as well as with enolase. Moreover, even expression of PKM2 is not enough to generate lactate as a driving fuel of carcinogenesis and tumor progression, because it seems that PKM2 is dependent on other cell signaling pathways through serine such as mTOR1 [225,226].

### 3. Lactate production

The tricarboxylic acid acid cycle (TCA) produces high amounts of protons that may decrease pH_i_. However, molecular oxygen interacts with protons to produce H_2_O. So, actually, oxygen acts as a “detoxifying” agent. Therefore, in the absence of oxygen, pyruvate is converted to lactate and takes NADH to produce NAD^+^ through lactate dehydrogenase activity, and so conversion into lactate may be a compensation to overcome cellular death.

Cancer cells adapt to hypoxia. Over-expression of HIF1-alpha induces pyruvate dehydrogenase kinase expression and that activates pyruvate dehydrogenase [227,228]; *i.e.* a shut-down of the mitochondria's role in glucose utilization. Thus, over-expression of proton transporters and over-expression of HIF1-alpha function as a strategic defense to prevent H^+^ accumulation. Although oxygen antagonizes hypoxic-induced proteins [229-231], cancer cells have a high rate of glycolysis even in the presence of oxygen [9]. Therefore, cancer cells prefer to increase LDH/PDH ratio.

## An integral perspective on the pH of cancer cells and the Warburg Effect: a synthetic explanation

Beyond the in-depth and dynamic consideration of each of the glycolytic steps, the idea that the shift to glycolytic metabolism relative to oxidative phosphorylation under aerobic conditions could be explained by an increase in the intracellular pH has been increasingly gaining weight with the passing of time [19,81-83,232,233]. Nagata et al., as well as our group have recently reached the conclusion that the Warburg effect can perhaps be fully explained by the simple elevation of pH_i_ in cancer cells [19,84]. These groups and others have also shown that malignant alkalinisation drives the initial activation of aerobic glycolysis (first appearance of the Warburg Effect) [10,19,176,234].

Thus, among all the many allosteric factors controlling glycolysis, the (H^+^), hydrogen ion concentration and/or pH, has become the most significant factor, even overwhelming all others (Table 1). In** the presence of adequate oxygen levels, the intracellular pH plays a key role in determining the way cancer cells obtain energy: an alkaline pH_i_ driving aerobic glycolysis and an acidic pH driving oxidative phosphorylation [233]. An explanation for this phenomenon derives from the fact that both the processes of OXPHOS and glycolysis are exquisitely but oppositely pH-sensitive, and a rapid shift of cell metabolic patterns follows either acidification or alkalinisation. In this vein, it has been known for decades that an alkaline pH_i_ even slightly above steady-state levels stimulates the activity of key glycolytic enzymes such as phosphofructokinase (PFK-1) and at the same time inhibits gluconeogenesis [5,80-83]. Indeed, the steady high pH_i_ characteristic of cancer cells can increase the allosteric regulation of PFK-1 more than 100-fold [52,82]. Indeed, it can now be considered that the high pH_i_ of tumor cells, the Warburg effect and the steady-state and selective hallmark of all cancer cells proton reversal may very well represent one and the same phenomenon observed from different perspectives, at different historical times and through less integral perspectives [8].

## The errors and limitations of Otto Warburg's theory. A further insight into the primary cause of cancer

To understand the meaning of the most recent and dynamic observations and interpretations on glycolysis we need to go back to the postulated origin of cancer cells by Warburg [1,235]. In doing so, we can realize that a fundamental confusion in the entire field of metabolic and biochemical cancer research was created from its very beginning.

Presently, it is clear that Otto Warburg was wrong on perhaps the main and most obscure point of his famous theory during his time, namely, the levels of cancer cell pH_i_ and, consequently, on its relationship to glycolysis. Indeed, Warburg always believed that the intracellular pH of cancer cells was acid because of their high production rates of lactic acid [236-238]. Probably, the main reason for overlooking the fundamental pH/glycolysis relationship, or at least for being given a secondary role at that time was that, during the 60's and 70's, the necessary technology to measure pH_i_ was not available [239]. This situation, however, started to turn around just after Warburg's death in 1970, when different reports began to emphasize that the pH_i_ of cancer cells was the opposite from what was generally thought during Warburg's life [83,232,239-241]. Thus, Warburg could not have been aware of the essential fact that cellular alkalosis not only activates glycolysis but at the same time hinders oxidative phosphorylation and the entrance of pyruvate in the Krebs cycle [81,242]. While this simple consideration may turn his theory completely upside down, at the same time it allows a further insight into the reasons behind decades of confusion and disagreements on his theory of “the abnormal respiratory mechanisms of cancer cells”, that he defended until his death in 1970 [2,81,82,233,235,242,243]. It is also important to remember that at Warburg's time there were not techniques permitting the discrimination between the pH of the cytosol and of the internal organelles. Today we are able to show that within tumor cells the cytosol is alkaline while the cytoplasmic vesicles are very acidic [244-246]. This is possible thanks to proton pumps and transporters, on one side eliminating protons outside the tumor cell when expressed on the plasma membrane, while pumping them from the cytosol into the internal lumen of the acidic vacuoles in order to avoid internal acidification [247].

Most importantly, any consideration concerning the intimate relationship of high pH_i_ and glycolysis was also fully missed during the famous arguments mainly between Otto Warburg and Sidney Weinhouse published in Science in 1956 [2,16]. Indeed, all those heated discussions could have been obviated if the true effect of pH on anaerobic and/or aerobic glycolysis and oxidative phosphorylation (“parahypoxia”) [89] could have been taken into account. Probably, this is also the main reason behind the fact that the search for the real cause underlying the Warburg effect has created many disagreements during the last decades [2,15,53,238,248-254]. All in all, it can now be said that Warburg was right up to a certain point but that his critics were also partially right. However, all of them missed the main point. Aerobic glycolysis or damaged respiration was not the primary cause of cancer, as Warburg defended all his life. Indeed, the primary cause of cancer appears to be, precisely, the main cause behind the aerobic glycolysis of tumors: a profound disruption of the homeostatic acid-balance of the cell represented by an abnormally high pH_i_ induced and maintained by an extremely varied number of etiological factors of different natures (for a review, see ref. No. 8).

In summary, cellular alkalosis represents a common final pathway in cell transformation induced by a myriad of different stimuli, from oncogenes to virus to mitogens to growth factors and hormones to gene products [5,8,10,11,82,84,88,176,255]. Finally, it is interesting to note that some recent and otherwise complete reviews dealing with Warburg's contributions to modern concepts in cancer metabolism, tumor glycolysis, the initiation of cancer and oxidative phosphorylation have not even considered the tight cause-effect interrelationships between intracellular and extracellular pH, glycolysis, the Warburg effect and cancer proton reversal [15,248,250].

## Anticancer and antimetastatic potential of the new and potent NHE1 inhibitors

The development and maintenance of a reversed pH gradient in cancer cells of all malignant tumors (high pH inside/low pH outside), which is the opposite to the normal situation, is accepted to be directly due to the ability of the tumor cells to secrete protons (H^+^) [11,84,255,256]. This proton secretion depends on the buffering capacity of the cell and is driven by a series of membrane-bound proton transporters (MBPT), mainly the Na^+^/H^+^ exchangers but also carbonic anhydrases (CAs, mainly CA IX and XII), vacuolar H^+^-ATPases, the H^+^/Cl^−^ symporter, the monocarboxylate transporter (MCT, mainly MCT1), also known as the lactate-proton symporter, the Na^+^-dependent Cl^−^/HCO_3_^−^ exchanger or bicarbonate transporter and the ATP synthase [11,85,256-260], each of them having its specific inhibitors [261].

Among them, the most important, functionally active, cancer-selective and better studied proton transporter is the Na^+^/H^+^ exchanger isoform 1, NHE1 [262-264]. The NHE1 is specifically involved in cellular acid-base balance and is the predominant isoform expressed in tumors, where it has been shown that it contributes to cellular pH homeostasis, cell transformation, proliferation, motility, migration, tumor growth, invasion, activation of the metastatic process, resistance to chemotherapy and probably also for at least certain cases of spontaneous regression of cancer [84,265-269]. An elevated NHE1 activity is considered to be the major factor in promoting tumor extracellular/interstitial acidity from even the earliest pre-cancer stage of oncogene-driven neoplastic transformation [176,270,271]. Regarding NHE-related malignant angiogenesis, the activity of a significant number of proangiogenic factors and oncogenes has been shown to positively affect NHE1 expression while, on the contrary, a wide array of anti-angiogenic drugs inhibit NHE1 [272,273]. Conversely, decreasing NHE1 expression or inhibiting NHE1 activity leads to acidification of the intracellular space and so to the inhibition of glycolysis, thus to tumour cell growth arrest and, finally, to selective apoptosis [234,267,274,275]. Consequently, the new, potent and highly selective NHE1 inhibitors - mainly Cariporide, Phx-3 and Compound 9t - appear predestined to be taken advantage of as a new and highly selective therapeutic “magic bullets” in probably most types of human cancer [51,263,276-278].

**Amiloride:** This compound was the first NHE inhibitor developed and it was shown to decrease vasoendothelial growth factor (VEGF) production and the activity of urokinase-type plasminogen activator (μPA), metalloproteinases (MMP) and other proteases, all of which aid in the activation of the metastatic process [277,279-282]. Amiloride alone was shown to achieve a complete *in vivo* anti-metastatic effect in transplanted tumors in rats [283]. Indeed, there are occasional reports of long-term treatment with Amiloride in humans achieving remissions of cancer after chemotherapy had failed to control disease progression [284]. Recent publications on the use of amiloride in cancer therapy discussed the different studies where its utilization had clear anti-neoplastic effects with few side-effects [285]. This potassium-sparing diuretic, apart from having a direct antitumoral, antimetastatic and antiangiogenic effect [283,285,286], at least in part by inhibiting uPA and VEGF, has been shown to be well tolerated and safe when used in the chronic situation in pharmacological dosages in humans, the main side-effect being occasionally increased plasma K^+^ levels [284,287,288]. Since more selective and powerful NHE inhibitors, like Cariporide, Phx-3 and compound 9t are not available for human use [19,289,290], amiloride should still be part of new protocols dealing with the concerted use of a cocktail of proton transport inhibitors (PTIs) as anticancer agents in different human solid tumors [85,284,291].

**HMA:** Along the same line, striking results in different kinds of leukemic cells were reported with the potent NHE1 inhibitor HMA (5-(N,N-hexametylene)-amiloride), which specifically decreases the pH_i_ well below the survival threshold leading to selective apoptosis in a variety of human leukemic cells [274]. This has led to the consideration that inducing a low pH_i_-mediated apoptosis as a cancer-specific therapeutic modality for all cancer cells and tissues could be a new and original approach to clinical therapeutics [19,89,255,257,292]. In summary, a great deal of evidence has been accumulating showing that the NHE1, among other MBPT (membrane-bound proton transporters) is an important, and possibly selective, anticancer target [89,263,276-278,288]. The pharmacology and therapeutic possibilities of the rest of the different proton transporters besides NHE1 have been thoroughly reviewed recently and will not be further dealt with here [84,260,263,269].

**Cariporide:** It has been demonstrated that treating various kinds of cancer cells with selective and potent inhibitors of NHE1, including Cariporide, suppresses their invasive capability [268,293-295]. Di Sario *et al.,* have also shown that Cariporide, through its selective inhibition of NHE1 and subsequent decrease of intracellular pH reduces proliferation and induces apoptosis in cholangiocarcinoma cells [296], leading these authors to suggest the potential therapeutic value of Cariporide against this human tumor. A recent review has also focused on how to therapeutically target the NHE1-mediated metabolic transformations of cancer cells with Cariporide [253]. The only non-Amiloride based compounds with NHE1 inhibitory activity that have undergone clinical trials are Cariporide and Eniporide, and, unfortunately, those trials were not in the field of cancer but in a cardiological setting and for ischaemic-reperfusion injury [297-301]. Cariporide has been shown to be useful in overcoming multiple drug resistance (MDR) and the activity of the metastatic process [302]. Besides, it is orally bioavailable and by this route of administration has been used but, unfortunately, never to date as an anticancer drug [297,299-301,303-308]. Cariporide also reduces hypoxia-mediated tumor invasion of human tongue squamous cell carcinoma by inhibiting NHE1 [309]. In this study, the authors demonstrated that inhibition of NHE1 by Cariporide (HOE-642) suppressed the invasion and migration of Tca8113 cells under hypoxic conditions. In another study pharmacological inhibition of p38 MAPK (mitogen-activated protein kinase) also significantly suppressed C/EBPα expression under hypoxia conditions after NHE1 inhibition [295]. Indeed, in addition to VEGF release and, subsequently, neoangiogenesis, being stimulated by hypoxia, upregulation of VEGF has also been linked as being secondary to acidic pH_e_ [310,311]. Also, NHE1-dependent lowering in pH_i_, apart from deactivating glycolysis at its different enzymatic targets also reduces the release of VEGF from the tumor cell so hindering motility and invasion [274,312].

**Phx-3:** An additional series of NHE1 inhibitors whose structure is independent of Amiloride have been later developed. Phx-3 (2-Aminophenoxazine-3-one) is highly selective for NHE1 inhibition and was shown to selectively stimulate apoptosis in a variety of cancer cell lines while normal lymphocytes were not affected [19,234]. Also, Phx-3 also effectively reversed a subcutaneously injected adult T-cell leukaemia tumor growth in animal studies without noticeable toxicity (A. Tomoda, personal communication).

**Compound 9t:** Otherwise, researchers at Bristol-Myers synthesized a 5-aryl-4-(4-(5-methyl-1*H*-imidazol-4-yl) piperididn-1-yl) pyrimidine analog (compound 9t) that was reported to have an excellent NHE1 inhibitory activity 500-fold more potent than cariporide. Besides, compound 9 has a reported 52% oral bioavailability, a plasma half-life of 1.5 hours in rats, low side-effects in mice and may possess a significantly improved safety profile over other NHE1 inhibitors [290]. Unfortunately, there have been no further publications utilizing this compound in any anticancer attempt either *in vitro* or *in vivo*.

Finally, there are many reasons to think that any of these new and potent NHE1 inhibitors could have a significant selectivity in the treatment of cancer, since even if NHE1 is ubiquitous and plays a fundamental role in pH housekeeping and volume control, it is also well known that in normal tissues the NHE1 is quiescent and is activated only during acidosis or cell shrinkage. Therefore, blocking it will have very little effect on normal tissues. This should be an advantage to consider and exploit as an important degree of specificity in the anticancer effect of NHE1 inhibitors, as it has been known from cell studies since the year 2000 [19,274].

However, during the last few years the holding of international patents on the new, selective and powerful NHE1 inhibitors by different pharmacological companies has made, and it is still making most difficult to achieve any real progress along these new and highly promising anticancer therapeutic lines, as it has been recently proposed [51,313]

## Discussion. Proton transport inhibition (PTI) as a selective tumor antiglycolytic and anticancer therapeutic approach. A new strategy after one hundred years of metabolic cancer research

The utilization of different proton transport inhibitors (PTIs) in cancer therapeutics was originally suggested by the group of Pouysségur and our group as a novel approach to the pH-related treatment of malignant tumors because of its potential as a more selective and less toxic approach to therapeutics than conventional chemotherapy [85,252,314]. Pouysségur has also proposed the use of PTIs as a valid approach to cancer treatment, advancing that this ‘pH-targeted’ therapy, perhaps combined with anti-angiogenesis in order to increase hypoxia-mediated acidosis, would synergistically induce the collapse and massive shrinkage of solid tumours [314]. Similarly, from the therapeutic point of view, inhibiting tumor glycolysis and reverting the Warburg effect by selective intracellular acidification has been advanced as a treatment of cancer [8,19]. Indeed, in the light of the older and the more recent contributions [5,19,84,233,234,242] it can now be concluded that counteracting the Warburg effect and its aerobic glycolysis through any therapeutic method directed to selectively induce intracellular acidification in cancer cells and/or reverting proton reversal now appears to represent one and the same phenomenon.

In summary, the most potent and promising Amiloride and non-Amiloride derivatives, such as Cariporide, Phx-3 and compound 9t [19,268,290,298] need to be included in pre-clinical and clinical trials as an important part of the anticancer armamentarium. That these compounds have not yet reached translational oncology becomes difficult to understand taking into account the massive theoretical background, available preclinical data as well as the results of the molecular, biochemical and metabolic studies already available at the present time. These anticancer compounds can be useful either as antitumoral and chemotherapeutic agents on their own, in the context of preventing and controlling the metastatic process and in any attempts to reverse MDR [8].

It is expected that the effects of a targeted therapy will not be durable when the therapy is designed to target a single enzyme or biological molecule. This is because cellular pathways operate like webs with multiple redundancies or alternate routes that may be activated in response to the inhibition of a certain pathway. For this reason, combination and concerted therapies with PTIs will be often needed to effectively treat many tumors screened for pertinent pathway dependence. It can be advanced that the new NHE1 inhibitors show a great promise as a new and selective approach to the treatment of a wide array of different malignant tumours and even leukaemias and, hopefully, they will help to overcome the present impasse and flat progress in cancer treatment [291,315,316]. These strategies have been recently discussed in an occasional review [8,84] and in a perspective [85], and introduce a real paradigm shift in cancer treatment. At the same time, there is a continuously growing interest in this new paradigm as shown by the number of its publications increasingly available in the most recent scientific literature (313, 318).

## CONCLUSIONS

The Warburg Effect represents an unusual strategy of cellular defense that reduces the oxidative stress status of the cells and so it has certain evolutionary advantages. This has made targeting of glycolytic enzymes a very appealing approach for decades, but unfortunately so far with dissapointing results. Designing any future strategy should take into account the crossing of drugs across the heterogeneous multi-habitats of cancer cells and tissues in order to cover all the tumor cell populations. In conclusion, before investing in the discovery of new pathways and introducing new biological techniques, a new approach to cancer therapeutics could be achieved by introducing novel and broadminded perspectives to the fight against human malignant tumors and leukaemias.

In this vein, even from the times of Walter Cannon and Hans Selye cell acid-base balance has been recognized to be the main parameter to define cellular homeostasis, the life of cells being possible only within a very narrow range of pH (less than one unit). It becomes essential to recognize that the pH of normal cells and cancer cells deviate towards opposite ends of a biological and metabolic spectrum. This energetic abnormality represents the largest difference among normal cellular physiology and cancer pathophysiology and a recently recognized new and selective hallmark of all cancer cells and tissues [8,176].

### Summary

From an etiological and etiopathogenic perspective, the hydrogen-related dynamics of malignancy has become a new approach to cancer and its dynamics and energetic mechanisms that is helping to reach a better understanding of several, until now disparaged areas of cancer research both at basic and clinical levels, as well as of the intimate nature of malignant disease. This unifying thermodynamic view now permits an integration of different cancer fields, ranging from cell transformation and metabolism, local growth and invasion, neovascularisation and the activation and progression of the metastatic process (“pH centric paradigm”).

From a therapeutic perspective, the primary aim of this pH-based approach to cancer treatment is to manipulate the selective forces controlling the dysregulated pH dynamics of all cancer cells and tissues in-order-to regress tumor growth, control local invasion and deactivate the metastatic potential of malignant tumors. All available evidence seems to indicate that this would take place regardless of pathological differences, tissue type or genetic origin. This therapeutic approach would also provide much less toxicity than present day treatments, probably also more effective therapies than any other chemotherapy known to date and it has real possibilities to become a successful strategy in treating human cancer in general. A pathologically elevated pH_i_ and its associated proton reversal (a reversed pH gradient in cancer cells and tissues (ΔpH_i_ to ΔpH_e_, ↑pH_i_/↓pH_e_) can perhaps be now considered the most specific cancer abnormality and essential hallmark of all kinds of malignant cells and tissues.

This hydrogen ion-based perspective has also permitted the better understanding of the Warburg effect, which can now be simply explained by the effects of the concerted action of proton transporters in increasing intracellular pH and stimulating aerobic glycolysis. In this respect, Otto Warburg and his contemporaries committed an important historical error that has possibly misled several decades of metabolic and biochemical cancer research. The main limitation was probably imposed by the lack of available intracellular pH measurements before the time of Warburg's death in 1970. We also conclude that the high pH_i_ of tumor cells, the Warburg effect and the proton reversal of cancer cells and tissues are likely to represent one and the same phenomenon defined in different ways.

Any attempt to therapeutically induce a selective intracellular acidification as a selective antiglycolytic treatment using proton transport inhibitors (PTIs) in all cancer cells and tissues would secondarily increase interstitial tumoral pH, thus inhibiting the metastatic process. This represents a rational and firmly based approach to cancer treatment at all its stages of development. Further, it has the potential of being selectively exploited in the treatment of many different malignancies.

Cariporide, other potent NHE1 inhibitors of the Amiloride series, as well as powerful and selective NHE1 inhibitors of the non-Amiloride series, like Phx-3 and compound 9t, have the potential of being highly promising, minimally toxic and truly effective anticancer agents in a wide array of malignant tumours and leukaemias, hopefully representing a new paradigm in cancer therapeutics.

In order to achieve significant progress along these new lines, a radical change of vision is strongly needed from the pharmacological companies that hold international patents on the new and selective NHE1 inhibitors and international legislations in these areas. This will be a great help furthering preclinical and cancer clinical research and treatment using proton transport inhibitors in modern and less toxic anticancer therapeutics.

## References

[1] Warburg O, Wind F, Negelein E (1927). The metabolism of tumors in the body. J Gen Phys.

[2] Warburg O (1930). The Metabolism of Tumors.

[3] Robey IF, Martin NK (2011). Bicarbonate and dichloroacetate: evaluating pH altering therapies in a mouse model for metastatic breast cancer. BMC Cancer.

[4] Cori CF, Cori G.T. (1925). The carbohydrate metabolism of tumors. II. Changes in the sugar, lactic acid, and co-combining power of blood passing through a tumor. J Biol Chem.

[5] Harguindey S, Henderson ES, Naeher C (1979). Effects of systemic acidification of mice with Sarcoma 180. Cancer Res.

[6] Harguindey S (1982). Hydrogen ion dynamics and cancer: an appraisal. Med Pediat Oncol.

[7] Harguindey S, Reshkin SJ, Orive G, Arranz JL, Anitua E (2007). Growth and trophic factors, pH and the Na+/H+ exchanger in Alzheimer's disease, other neurodegenerative diseases and cancer: new therapeutic possibilities and potential dangers. Current Alzh Res.

[8] Harguindey S, Arranz JL, Polo Orozco JD, Rauch C, Fais S, Cardone RA (2013). Cariporide and other new and powerful NHE1 inhibitors as potentially selective anticancer drugs - an integral molecular/biochemical/metabolic/clinical approach after one hundred years of cancer research. J Transl Med.

[9] Alfarouk KO, Shayoub MEAA, Muddathir AK, Elhassan GO, Bashir AHHH (2011). Evolution of Tumor Metabolism might Reflect Carcinogenesis as a Reverse Evolution process (Dismantling of Multicellularity). Cancers.

[10] Reshkin SJ, Bellizzi A, Caldeira S, Albarani V, Malanchi I, Poignee M (2000). Na+/H+ exchanger-dependent intracellular alkalinization is an early event in malignant transformation and plays an essential role in the development of subsequent transformation-associated phenotypes. FASEB J.

[11] Cardone RA, Casavola V, Reshkin SJ (2005). The role of disturbed pH dynamics and the Na+/H+ exchanger in metastasis. Nature Revs Cancer.

[12] Jang M, Kim SS, Lee J (2013). Cancer cell metabolism: implications for therapeutic targets. Experim Mol Med.

[13] Hanahan D, Weinberg RA (2011). Hallmarks of cancer: the next generation. Cell.

[14] Racker E (1974). History of the Pasteur effect and its pathobiology. Molecular Cell Biochem.

[15] Burk D, Woods M, Hunter J (1967). On the significance of glucolysis for cancer growth, with special reference to Morris rat hepatomas. J Nat Cancer Instit.

[16] Weinhouse S (1956). On respiratory impairment in cancer cells. Science.

[17] Alfarouk KO, Bashir AHH (2011). Diabetes mellitus type 2 through oncology lens. Med Hypotheses.

[18] Onodera Y, Nam J-M, Bissell MJ (2014). Increased sugar uptake promotes oncogenesis via EPAC/RAP1 and O-GlcNAc pathways. J Clinical Invest.

[19] Nagata H, Che X-F, Miyazawa K, Tomoda A, Konishi M, Ubukata H (2011). Rapid decrease of intracellular pH associated with inhibition of Na+/H+ exchanger precedes apoptotic events in the MNK45 and MNK74 gastric cancer cell lines treated with 2-aminophenoxazine-3-one. Oncol Rep.

[20] Lane AN, Fan TW-M, Higashi RM (2008). Metabolic acidosis and the importance of balanced equations. Metabolomics.

[21] Racker E (1981). Warburg effect revisited. Science.

[22] Ritter P (1996). Biochemistry A foundation.

[23] Robey RB, Hay N (2005). Mitochondrial hexokinases: guardians of the mitochondria. Cell Cycle (Georgetown, Tex).

[24] Bustamante E, Pedersen PL (1977). High aerobic glycolysis of rat hepatoma cells in culture: role of mitochondrial hexokinase. PNAS USA.

[25] Casneuf VF, Fonteyne P, Van Damme N, Demetter P, Pauwels P, de Hemptinne B (2008). Expression of SGLT1, Bcl-2 and p53 in primary pancreatic cancer related to survival. Cancer Invest.

[26] Vera JC, Reyes AM, Velásquez F V, Rivas CI, Zhang RH, Strobel P (2001). Direct inhibition of the hexose transporter GLUT1 by tyrosine kinase inhibitors. Biochemistry.

[27] Young CD, Lewis AS, Rudolph MC, Ruehle MD, Jackman MR, Yun UJ (2011). Modulation of glucose transporter 1 (GLUT1) expression levels alters mouse mammary tumor cell growth <italic>in vitro</italic> and <italic>in vivo</italic>. PloS one.

[28] Wood TE, Dalili S, Simpson CD, Hurren R, Mao X, Saiz FS (2008). A novel inhibitor of glucose uptake sensitizes cells to FAS-induced cell death. Molecular Cancer Therap.

[29] Chen W, Guéron M The inhibition of bovine heart hexokinase by 2-deoxy-D-glucose-6-phosphate: characterization by 31P NMR and metabolic implications. Biochimie.

[30] Hoerter J, Dormont D, Girault M, Guéron M, Syrota A (1991). Insulin increases the rate of degradation of 2-deoxy-glucose-6-phosphate in the perfused rat heart: a 31P NMR study. J Mol Cell Cardiol.

[31] Hansen P, Gulve E, Gao J, Schluter J, Mueckler M, Holloszy J (1995). Kinetics of 2-deoxyglucose transport in skeletal muscle: effects of insulin and contractions. American J Physiol.

[32] Bertoni JM (1981). Competitive inhibition of rat brain hexokinase by 2-deoxyglucose, glucosamine, and metrizamide. J Neurochem.

[33] Bertoni JM (1982). Metrizamide inhibits human brain hexokinase. Neurology.

[34] Rempel A, Mathupala SP, Griffin CA, Hawkins AL, Pedersen PL (1996). Glucose catabolism in cancer cells: amplification of the gene encoding type II hexokinase. Cancer Res.

[35] Peng Q, Zhou Q, Zhou J, Zhong D, Pan F, Liang H (2008). Stable RNA interference of hexokinase II gene inhibits human colon cancer LoVo cell growth *in vitro* and *in vivo*. Cancer Biol Ther.

[36] Sweet IR, Li G, Najafi H, Berner D, Matschinsky FM (1996). Effect of a glucokinase inhibitor on energy production and insulin release in pancreatic islets. Am J Physiol.

[37] Alfarouk KO, Ibrahim ME, Gatenby R a, Brown JS (2013). Riparian ecosystems in human cancers. Evolut Appl.

[38] Ghosh A, Shieh J-J, Pan C-J, Sun M-S, Chou JY (2002). The catalytic center of glucose-6-phosphatase. HIS176 is the nucleophile forming the phosphohistidine-enzyme intermediate during catalysis. J Biol Chem.

[39] Register TC, Wuthier RE (1984). Effect of vanadate, a potent alkaline phosphatase inhibitor, on 45Ca and 32Pi uptake by matrix vesicle-enriched fractions from chicken epiphyseal cartilage. J Biol Chem.

[40] Weber G, Cantero A (1955). Glucose-6-phosphatase activity in normal, pre-cancerous, and neoplastic tissues. Cancer Res.

[41] Hadjiolov D (1969). Glucose-6-phosphatase activity in primary rat liver tumors induced by high doses of 4-dimethylaminoazo-benzene. Zeit für Krebs.

[42] Furuya T, Matsumoto I, Tsuchiya N, Hakoda M, Ichikawa N, Yago T Anti-glucose-6-phosphate isomerase, anti-cyclic citrullinated peptide antibodies and HLA-DRB1 genotypes in Japanese patients with early rheumatoid arthritis. Clinical Exp Rheumatol.

[43] Hayashi T, Matsumoto I, Muraki Y, Takahashi R, Chino Y, Goto D (2005). Clinical characteristics of anti-glucose-6-phosphate isomerase antibody-positive Japanese patients with rheumatoid arthritis. Mod Rheumatol-Japan Rheumat Assoc.

[44] Schaller M, Stohl W, Tan SM, Benoit VM, Hilbert DM, Ditzel HJ (2005). Raised levels of anti-glucose-6-phosphate isomerase IgG in serum and synovial fluid from patients with inflammatory arthritis. Ann Rheum Dis.

[45] Allavena P, Garlanda C, Borrello MG, Sica A, Mantovani A (2008). Pathways connecting inflammation and cancer. Curr Opin Genet Dev.

[46] Rakoff-Nahoum S (2006). Why cancer and inflammation?. Yale J Biol Med.

[47] Faik P, Walker JI, Redmill AA, Morgan MJ (1988). Mouse glucose-6-phosphate isomerase and neuroleukin have identical 3′ sequences. Nature.

[48] Watanabe H, Takehana K, Date M, Shinozaki T, Raz A (1996). Tumor cell autocrine motility factor is the neuroleukin/phosphohexose isomerase polypeptide. Cancer Res.

[49] Yanagawa T, Funasaka T, Tsutsumi S, Watanabe H, Raz A (2004). Novel roles of the autocrine motility factor/phosphoglucose isomerase in tumor malignancy. Endocrine-related Cancer.

[50] Toyoda Y, Miwa I, Ogiso S, Okuda J (1993). Two interconvertible forms of glucose-6-phosphate isomerase in rat muscle. Biochem Mol Biol Internat.

[51] Huber V, De Milito A, Harguindey S, Reshkin SJ, Wahl ML, Rauch C (2010). Proton dynamics in cancer. J Transl Med.

[52] Webb BA, Chimenti M, Jacobson MP, Barber DL (2011). Dysregulated pH: a perfect storm for cancer progression. Nature Revs Cancer.

[53] Neri D, Supuran CT (2011). Interfering with pH regulation in tumours as a therapeutic strategy. Nature Revs Drug Discov.

[54] Tsutsumi S, Hogan V, Nabi IR, Raz A (2003). Overexpression of the autocrine motility factor/phosphoglucose isomerase induces transformation and survival of NIH-3T3 fibroblasts. Cancer Res.

[55] Liotta LA, Mandler R, Murano G, Katz DA, Gordon RK, Chiang PK (1986). Tumor cell autocrine motility factor. PNAS USA.

[56] Torimura T, Ueno T, Kin M, Harada R, Nakamura T, Kawaguchi T (2001). Autocrine motility factor enhances hepatoma cell invasion across the basement membrane through activation of beta1 integrins. Hepatology (Baltimore, Md).

[57] Funasaka T, Hogan V, Raz A (2009). Phosphoglucose isomerase/autocrine motility factor mediates epithelial and mesenchymal phenotype conversions in breast cancer. Cancer Res.

[58] Tsutsumi S, Fukasawa T, Yamauchi H, Kato T, Kigure W, Morita H (2009). Phosphoglucose isomerase enhances colorectal cancer metastasis. Internat J Oncol.

[59] Speranza ML, Valentini G, Malcovati M (1990). Fructose-1,6-bisphosphate-activated pyruvate kinase from Escherichia coli. Nature of bonds involved in the allosteric mechanism. Europ J Biochemist/FEBS.

[60] Jurica MS, Mesecar A, Heath PJ, Shi W, Nowak T, Stoddard BL (1998). The allosteric regulation of pyruvate kinase by fructose-1,6-bisphosphate. Structure.

[61] Bajić A, Zakrzewska J, Godjevac D, Andjus P, Jones DR, Spasić M (2011). Relevance of the ability of fructose 1,6-bis(phosphate) to sequester ferrous but not ferric ions. Carbohydrate Res.

[62] Planas ME, Sánchez S, González P, Rodrigues de Oliveira J, Bartrons R (1993). Protective effect of fructose 1,6-bisphosphate against carrageenan-induced inflammation. Europ J Pharmacol.

[63] Sola A, Berrios M, Sheldon RA, Ferriero DM, Gregory GA (1996). Fructose-1,6-bisphosphate after hypoxic ischemic injury is protective to the neonatal rat brain. Brain Res.

[64] Edde L, Zhou X, Eaton JW, Sherman MP (1998). Induction of nitric oxide synthase in macrophages: inhibition by fructose-1,6-diphosphate. Biochem Biophys Res Comm.

[65] Ahn SM, Yoon H-Y, Lee BG, Park KC, Chung JH, Moon C-H (2002). Fructose-1,6-diphosphate attenuates prostaglandin E2 production and cyclo-oxygenase-2 expression in UVB-irradiated HaCaT keratinocytes. British J Pharmacol.

[66] Balendiran GK, Dabur R, Fraser D The role of glutathione in cancer. Cell Biochem Function.

[67] Vexler ZS, Wong A, Francisco C, Manabat C, Christen S, Täuber M (2003). Fructose-1,6-bisphosphate preserves intracellular glutathione and protects cortical neurons against oxidative stress. Brain Res.

[68] Cohly H, Jenkins J, Skelton T, Meydrech E, Markov AK (2004). Fructose-1,6-diphosphate suppresses T-lymphocyte proliferation, promotes apoptosis and inhibits interleukins-1, 6, beta-actin mRNAs, and transcription factors expression. Immunol Investigat.

[69] Coussens LM, Werb Z Inflammation and cancer. Nature.

[70] Tsai MY, Kemp RG (1973). Isozymes of rabbit phosphofructokinase. Electrophoretic and immunochemical studies. J Biol Chem.

[71] Vora S, Oskam R, Staal GE (1985). Isoenzymes of phosphofructokinase in the rat. Demonstration of the three non-identical subunits by biochemical, immunochemical and kinetic studies. Biochem J.

[72] Dunaway GA, Kasten T (1986). Are the rat tissue/organ proportions of 6-phosphofructo-1-kinase subunits strain-specific?. Biochem J.

[73] Dunaway GA, Kasten TP, Sebo T, Trapp R (1988). Analysis of the phosphofructokinase subunits and isoenzymes in human tissues. Biochem J.

[74] Zancan P, Sola-Penna M, Furtado CM, Da Silva D (2010). Differential expression of phosphofructokinase-1 isoforms correlates with the glycolytic efficiency of breast cancer cells. Mol Genet Metabol.

[75] Nakamura K, Kituta T, Nakamura Y, Nakajima Y, Kobayashi K, Uchida T (1987). PFK inhibition test for cancer detection: clinical applications and mechanisms of PFK inhibition. Cancer Detect Prevent.

[76] Babul J, Robinson JP, Fraenkel DG (1977). Are the aerobic and anaerobic phosphofructokinases of Escherichia coli different?. Europ J Biochem/FEBS.

[77] Andrés V, Carreras J, Cussó R (1990). Regulation of muscle phosphofructokinase by physiological concentrations of bisphosphorylated hexoses: effect of alkalinization. Biochem Biophys Res Commun.

[78] Erecińska M, Deas J, Silver IA (1995). The effect of pH on glycolysis and phosphofructokinase activity in cultured cells and synaptosomes. J Neurochem.

[79] Frieden C, Gilbert HR, Bock PE (1976). Phosphofructokinase. III. Correlation of the regulatory kinetic and molecular properties of the rabbit muscle enzyme. J Biol Chem.

[80] Trivedi B, Danforth WH (1966). Effect of pH on the kinetics of frog muscle phosphofructokinase. J Biol Chem.

[81] Relman AS (1972). Metabolic consequences of acid-base disorders. Kidney Internat.

[82] Rubin DF H (1974). Interrelationships of sugar transport and the initiation of DNA synthesisin chick embryo cells. Control of Proliferation of Animal Cells. Cold Spring Harbour Labs.

[83] Kaminskas E (1978). The pH-dependence of sugar-transport and glycolysis in cultured Ehrlich ascites-tumour cells. Biochem J.

[84] Reshkin SJ, Cardone RA, Harguindey S (2013). Na+-H+ exchanger, pH regulation and cancer. Recent Patents Anti-cancer Drug Discover.

[85] Harguindey S, Arranz JL, Wahl ML, Orive G, Reshkin SJ (2009). Proton transport inhibitors as potentially selective anticancer drugs. Anticancer Res.

[86] Brisson L, Reshkin SJ, Goré J, Roger S (2012). PH regulators in invadosomal functioning: Proton delivery for matrix tasting. Eur J Cell Biol.

[87] Brisson L, Gillet L, Calaghan S, Besson P, Le Guennec J-Y, Roger S (2011). Na(V)1.5 enhances breast cancer cell invasiveness by increasing NHE1-dependent H(+) efflux in caveolae. Oncogene.

[88] Perona R, Serrano R (1988). Increased pH and tumorigenicity of fibroblasts expressing a yeast proton pump. Nature.

[89] Harguindey S, Pedraz JL, García, Cañero R, Pérez de Diego J, Cragoe EJ (1995). Hydrogen ion-dependent oncogenesis and parallel new avenues to cancer prevention and treatment using a H(+)-mediated unifying approach: pH-related and pH-unrelated mechanisms. Critical Revs Oncogenesis.

[90] Colombo G, Tate PW, Girotti AW, Kemp RG (1975). Interaction of inhibitors with muscle phosphofructokinase. J Biol Chem.

[91] Blangy D, Buc H, Monod J (1968). Kinetics of the allosteric interactions of phosphofructokinase from Escherichia coli. J Mol Biol.

[92] Evans PR, Farrants GW, Hudson PJ (1981). Phosphofructokinase: structure and control. Philosophical transactions Royal Society of London Series B, Biological sciences.

[93] Shirakihara Y, Evans PR (1988). Crystal structure of the complex of phosphofructokinase from Escherichia coli with its reaction products. J Mol Biol.

[94] Wegener G, Krause U (2002). Different modes of activating phosphofructokinase, a key regulatory enzyme of glycolysis, in working vertebrate muscle. Biochem Soc Transact.

[95] Furtado CM, Marcondes MC, Sola-Penna M, de Souza MLS, Zancan P (2012). Clotrimazole preferentially inhibits human breast cancer cell proliferation, viability and glycolysis. PLoS ONE.

[96] el-Maghrabi MR, Lange AJ, Jiang W, Yamagata K, Stoffel M, Takeda J (1995). Human fructose-1,6-bisphosphatase gene (FBP1): exon-intron organization, localization to chromosome bands 9q22.2-q22.3, and mutation screening in subjects with fructose-1,6-bisphosphatase deficiency. Genomics.

[97] Herzog B, Waltner-Law M, Scott DK, Eschrich K, Granner DK (2000). Characterization of the human liver fructose-1,6-bisphosphatase gene promoter. Biochem J.

[98] Tillmann H, Eschrich K (1998). Isolation and characterization of an allelic cDNA for human muscle fructose-1,6-bisphosphatase. Gene.

[99] Tillmann H, Stein S, Liehr T, Eschrich K (2000). Structure and chromosomal localization of the human and mouse muscle fructose-1,6-bisphosphatase genes. Gene.

[100] Tillmann H, Bernhard D, Eschrich K (2002). Fructose-1,6-bisphosphatase genes in animals. Gene.

[101] el-Maghrabi MR, Lange AJ, Kümmel L, Pilkis SJ (1991). The rat fructose-1,6-bisphosphatase gene. Structure and regulation of expression. J Biol Chem.

[102] Taketa K, Pogell BM (1965). Allosteric Inhbition of rat liver fructose 1,6 -diphosphatase by adensoine 5′-monophosphate. J Biol Chem.

[103] Pontremoli S (1971). BLH. Fructose 1, 6-diphosphatases. Enzymes.

[104] Pilkis S, El-Maghrabi M, Pilkis J, Claus T (1981). Inhibition of fructose-1,6-biphosphatase by fructose 2,6-biphosphate. J Biol Chem.

[105] Schaftingen E V (1981). Inhibition of Fructose-1, 6-biphosphatase by Fructose 2,6-bisphosphate. PNAS USA.

[106] Myung S, Wang Y, Zhang Y-HP (2010). Fructose-1,6-bisphosphatase from a hyper-thermophilic bacterium Thermotoga maritima: Characterization, metabolite stability, and its implications. Process Biochem.

[107] Voehringer DW, Hirschberg DL, Xiao J, Lu Q, Roederer M, Lock CB (2000). Gene microarray identification of redox and mitochondrial elements that control resistance or sensitivity to apoptosis. PNAS USA.

[108] Alfarouk KO, Muddathir AK, Shayoub MEA (2011). Tumor acidity as evolutionary spite. Cancers.

[109] Becker J, Klopprogge C, Zelder O, Heinzle E, Wittmann C (2005). Amplified expression of fructose 1,6-bisphosphatase in Corynebacterium glutamicum increases *in vivo* flux through the pentose phosphate pathway and lysine production on different carbon sources. Applied Envir Microbiol.

[110] Van Schaftingen E, Hue L, Hers HG (1980). Control of the fructose-6-phosphate/fructose 1,6-bisphosphate cycle in isolated hepatocytes by glucose and glucagon. Role of a low-molecular-weight stimulator of phosphofructokinase. Biochemical J.

[111] Van Schaftingen E, Hue L, Hers HG (1980). Fructose 2,6-bisphosphate, the probably structure of the glucose- and glucagon-sensitive stimulator of phosphofructokinase. Biochem J.

[112] Thompson JE, Thompson CB (2004). Putting the rap on Akt. J Clin Oncol.

[113] Hue L, Rider MH (1987). Role of fructose 2,6-bisphosphate in the control of glycolysis in mammalian tissues. Biochem J.

[114] Wu C, Okar DA, Newgard CB, Lange AJ (2002). Increasing fructose 2,6-bisphosphate overcomes hepatic insulin resistance of type 2 diabetes. American J Physiol.

[115] Hue L, Maisin L, Rider MH (1988). Palmitate inhibits liver glycolysis. Involvement of fructose 2,6-bisphosphate in the glucose/fatty acid cycle. Biochem J.

[116] Levey GS (1975). The glucagon receptor and adenylate cyclase. Metabolism.

[117] Levey GS, Fletcher MA, Klein I (1975). Glucagon and adenylate cyclase: binding studies and requirements for activation. Advances Cyclic Nucleotide Res.

[118] Stewart HB, el-Maghrabi MR, Pilkis SJ (1986). Mechanism of activation of fructose-2,6-bisphosphatase by cAMP-dependent protein kinase. J Biol Chem.

[119] Perez JX, Roig T, Manzano A, Dalmau M, Boada J, Ventura F (2000). Overexpression of fructose 2,6-bisphosphatase decreases glycolysis and delays cell cycle progression. American J Physiol Cell Physiol.

[120] Pilkis SJ, Claus TH, Kurland IJ, Lange AJ (1995). 6-Phosphofructo-2-kinase/fructose-2,6-bisphosphatase: a metabolic signaling enzyme. Annual Rev Biochem.

[121] Smith WE, Langer S, Wu C, Baltrusch S, Okar DA (2007). Molecular coordination of hepatic glucose metabolism by the 6-phosphofructo-2-kinase/fructose-2,6- bisphosphatase:glucokinase complex. Mol Endocrin (Baltimore, Md).

[122] Wang Q, Donthi R V, Wang J, Lange AJ, Watson LJ, Jones SP (2008). Cardiac phosphatase-deficient 6-phosphofructo-2-kinase/fructose-2,6-bisphosphatase increases glycolysis, hypertrophy, and myocyte resistance to hypoxia. Am J Physiol Heart Circulat Physiol.

[123] Donthi R V, Ye G, Wu C, McClain DA, Lange AJ, Epstein PN (2004). Cardiac expression of kinase-deficient 6-phosphofructo-2-kinase/fructose-2,6-bisphosphatase inhibits glycolysis, promotes hypertrophy, impairs myocyte function, and reduces insulin sensitivity. J Biol Chem.

[124] Tornheim K (1985). Activation of muscle phosphofructokinase by fructose 2,6-bisphosphate and fructose 1,6-bisphosphate is differently affected by other regulatory metabolites. J Biol Chem.

[125] Bertrand L, Horman S, Beauloye C, Vanoverschelde J-L (2008). Insulin signalling in the heart. Cardiovasc Res.

[126] Xu J, Oshima T, Yoshida M (1991). Phosphoenolpyruvate-insensitive phosphofructokinase isozyme from Thermus thermophilus HB8. J Biochem.

[127] Berg J. M., Tymoczko J. L., S L (2002). Biochemistry.

[128] Rider MH, Crepin KM, De Cloedt M, Bertrand L, Hue L (1994). Site-directed mutagenesis of rat muscle 6-phosphofructo-2-kinase/fructose-2,6-bisphosphatase: role of Asp-130 in the 2-kinase domain. Biochem J.

[129] Okar DA, Manzano A, Navarro-Sabatè A, Riera L, Bartrons R, Lange AJ (2001). PFK-2/FBPase-2: maker and breaker of the essential biofactor fructose-2,6-bisphosphate. Trends Biochem Sci.

[130] Duran J, Gómez M, Navarro-Sabate A, Riera-Sans L, Obach M, Manzano A (2008). Characterization of a new liver- and kidney-specific pfkfb3 isozyme that is downregulated by cell proliferation and dedifferentiation. Biochem Biophys Res Commun.

[131] Clem B, Telang S, Clem A, Yalcin A, Meier J, Simmons A (2008). Small-molecule inhibition of 6-phosphofructo-2-kinase activity suppresses glycolytic flux and tumor growth. Mol Cancer Therap.

[132] Ros S, Santos CR, Moco S, Baenke F, Kelly G, Howell M (2012). Functional metabolic screen identifies 6-phosphofructo-2-kinase/fructose-2,6-biphosphatase 4 as an important regulator of prostate cancer cell survival. Cancer Discov.

[133] Minchenko O, Opentanova I, Minchenko D, Ogura T, Esumi H (2004). Hypoxia induces transcription of 6-phosphofructo-2-kinase/fructose-2,6-biphosphatase-4 gene via hypoxia-inducible factor-1alpha activation. FEBS letters.

[134] Cocco P (1987). Does G6PD deficiency protect against cancer? A critical review. J Epidemiol Community Health.

[135] Pilkis SJ, Chrisman TD, El-Maghrabi MR, Colosia A, Fox E, Pilkis J (1983). The action of insulin on hepatic fructose 2,6-bisphosphate metabolism. J Biol Chem.

[136] Winder WW, Carling JM, Duan C, Jones JP, Palmer SL, Walker MC (1994). Muscle fructose-2,6-bisphosphate and glucose-1,6-bisphosphate during insulin-induced hypoglycemia. J Applied Physiol.

[137] Sauvage M, Mazière P, Fathallah H, Giraud F (2000). Insulin stimulates NHE1 activity by sequential activation of phosphatidylinositol 3-kinase and protein kinase C zeta in human erythrocytes. Europ J Biochem./FEBS.

[138] Robert J, Van Rymenant M, Lagae F Enzymes in cancer III. Triosephosphate isomerase activity of human blood serum in normal individuals and in individuals with various pathological conditions. Cancer.

[139] Zhang X, Xiao Z, Li C, Xiao Z, Yang F, Li D (2009). Triosephosphate isomerase and peroxiredoxin 6, two novel serum markers for human lung squamous cell carcinoma. Cancer Sci.

[140] Wang X, Lu Y, Yang J, Shi Y, Lan M, Liu Z (2008). Identification of triosephosphate isomerase as an anti-drug resistance agent in human gastric cancer cells using functional proteomic analysis. J Cancer Res Clinic Oncol.

[141] Plaut B, Knowles JR (1972). pH-dependence of the triose phosphate isomerase reaction. Biochem J.

[142] Jang M, Kang HJ, Lee SY, Chung SJ, Kang S, Chi SW (2009). Glyceraldehyde-3-phosphate, a glycolytic intermediate, plays a key role in controlling cell fate via inhibition of caspase activity. Mol Cells.

[143] Penhoet E, Rajkumar T, Rutter WJ (1966). Multiple forms of fructose diphosphate aldolase in mammalian tissues. PNAS USA.

[144] Wachsmuth ED, Thöner M, Pfleiderer G (1975). The cellular distribution of aldolase isozymes in rat kidney and brain determined in tissue sections by the immuno-histochemical method. Histochem.

[145] Thompson RJ, Kynoch PA, Willson VJ (1982). Cellular localization of aldolase C subunits in human brain. Brain Res.

[146] Agius L (1996). Substrate modulation of aldolase B binding in hepatocytes. Biochem J.

[147] Lebherz HG, Rutter WJ (1969). Distribution of fructose diphosphate aldolase variants in biological systems. Biochem.

[148] Asaka M, Alpert E (1983). Subunit-specific radioimmunoassay for aldolase A, B, and C subunits: clinical significance. Ann New York Acad Sci.

[149] Sibley JA, Fleisher GA (1955). Aldolase content of normal and neoplastic tissue. Cancer Res.

[150] Asaka M, Kimura T, Meguro T, Kato M, Kudo M, Miyazaki T (1994). Alteration of aldolase isozymes in serum and tissues of patients with cancer and other diseases. J Clin Lab Anal.

[151] Ertunga NS, Colak A, Belduz AO, Canakci S, Karaoglu H, Sandalli C (2007). Cloning, expression, purification and characterization of fructose-1,6-bisphosphate aldolase from Anoxybacillus gonensis G2. J Biochem.

[152] Schapira F (1966). Aldolase isozymes in cancer. Eur J Cancer.

[153] Zamora-León SP, Golde DW, Concha II, Rivas CI, Delgado-López F, Baselga J, Nualart F, Vera JC (1996). Expression of the fructose transporter GLUT5 in human breast cancer. PNAS USA.

[154] Douard V, Ferraris RP (2008). Regulation of the fructose transporter GLUT5 in healthand disease. Am J Physiol Endocrin Metab.

[155] Berry MD, Boulton AA (2000). Glyceraldehyde-3-phosphate dehydrogenase and apoptosis. J Neurosci Res.

[156] Higashimura Y, Nakajima Y, Yamaji R, Harada N, Shibasaki F, Nakano Y (2011). Up-regulation of glyceraldehyde-3-phosphate dehydrogenase gene expression by HIF-1 activity depending on Sp1 in hypoxic breast cancer cells. Archiv Biochem Biophys.

[157] Hara MR, Thomas B, Cascio MB, Bae B-I, Hester LD, Dawson VL (2006). Neuroprotection by pharmacologic blockade of the GAPDH death cascade. PNAS USA.

[158] Ishitani R, Tajima H, Takata H, Tsuchiya K, Kuwae T, Yamada M (2003). Proapoptotic protein glyceraldehyde-3-phosphate dehydrogenase: a possible site of action of antiapoptotic drugs. Progress Neuro-psychopharm Psychiatr.

[159] Kragten E, Lalande I, Zimmermann K, Roggo S, Schindler P, Muller D (1998). Glyceraldehyde-3-phosphate dehydrogenase, the putative target of the antiapoptotic compounds CGP 3466 and R-(−)-deprenyl. J Biol Chem.

[160] Sagot Y, Toni N, Perrelet D, Lurot S, King B, Rixner H (2000). An orally active anti-apoptotic molecule (CGP 3466B) preserves mitochondria and enhances survival in an animal model of motoneuron disease. British J. Pharmacol.

[161] Carlile GW, Chalmers-Redman RM, Tatton NA, Pong A, Borden KE, Tatton WG (2000). Reduced apoptosis after nerve growth factor and serum withdrawal: conversion of tetrameric glyceraldehyde-3-phosphate dehydrogenase to a dimer. Molec Pharm.

[162] Ralser M, Wamelink MM, Kowald A, Gerisch B, Heeren G, Struys EA (2007). Dynamic rerouting of the carbohydrate flux is key to counteracting oxidative stress. J Biol.

[163] Vincent AM, TenBroeke M, Maiese K (1999). Neuronal intracellular pH directly mediates nitric oxide-induced programmed cell death. J Neurobiology.

[164] Fang B, Wang D, Huang M, Yu G, Li H (2010). Hypothesis on the relationship between the change in intracellular pH and incidence of sporadic Alzheimer's disease or vascular dementia. Internat J Neurosci.

[165] Sakai A, Shimizu H, Kono K, Furuya E (2005). Monochloroacetic acid inhibits liver gluconeogenesis by inactivating glyceraldehyde-3-phosphate dehydrogenase. Chemical Res Toxicol.

[166] Mountassif D, Baibai T, Fourrat L, Moutaouakkil A, Iddar A, El Kebbaj MS (2009). Immunoaffinity purification and characterization of glyceraldehyde-3-phosphate dehydrogenase from human erythrocytes. Acta Biochem Biophs Sinica.

[167] Blüthmann H, Cicurel L, Kuntz GW, Haedenkamp G, Illmensee K (1982). Immunohistochemical localization of mouse testis-specific phosphoglycerate kinase (PGK-2) by monoclonal antibodies. The EMBO Journal.

[168] Vandeberg JL, Lee CY, Goldberg E (1981). Immunohistochemical localization of phosphoglycerate kinase isozymes in mouse testes. J Experiment Zool.

[169] Wu S, Storey JM, Storey KB (2009). Phosphoglycerate kinase 1 expression responds to freezing, anoxia, and dehydration stresses in the freeze tolerant wood frog, Rana sylvatica. J Experiment Zool. Part A, Ecological Genetics Physiol.

[170] Zieker D, Königsrainer I, Weinreich J, Beckert S, Glatzle J, Nieselt K (2010). Phosphoglycerate kinase 1 promoting tumor progression and metastasis in gastric cancer - detected in a tumor mouse model using positron emission tomography/magnetic resonance imaging. Cell Physiol Biochem Pharm.

[171] Tang S-J, Ho M-Y, Cho H-C, Lin Y-C, Sun G-H, Chi K-H (2008). Phosphoglycerate kinase 1-overexpressing lung cancer cells reduce cyclooxygenase 2 expression and promote anti-tumor immunity *in vivo*. Intern J Cancer.

[172] Vassalli JD, Sappino AP, Belin D (1991). The plasminogen activator/plasmin system. J Clin Invest.

[173] Shetty S, Ganachari M, Liu M-C, Azghani A, Muniyappa H, Idell S (2005). Regulation of urokinase receptor expression by phosphoglycerate kinase is independent of its catalytic activity. American J Physiol Lung Cell Mol Physiol.

[174] Krietsch WK, Bücher T (1970). 3-phosphoglycerate kinase from rabbit sceletal muscle and yeast. European J Biochem./FEBS.

[175] Nojima H, Oshima T, Noda H (1979). Purification and properties of phosphoglycerate kinase from Thermus thermophilus strain HB8. J Biochem.

[176] Reshkin SJ, Greco MR, Cardone RA (2014). Role of pHi, and proton transporters in oncogene-driven neoplastic transformation. Phil Trans Royal Soc.,. London Series B, Biological Sciences.

[177] Ren F, Wu H, Lei Y, Zhang H, Liu R, Zhao Y (2010). Quantitative proteomics identification of phosphoglycerate mutase 1 as a novel therapeutic target in hepatocellular carcinoma. Mol Cancer.

[178] Chander M, Setlow B, Setlow P (1998). The enzymatic activity of phosphoglycerate mutase from gram-positive endospore-forming bacteria requires Mn2+ and is pH sensitive. Canad J Microbiol.

[179] Pancholi V (2001). Multifunctional alpha-enolase: its role in diseases. Cell Mol Life Sci.

[180] Ejeskär K, Krona C, Carén H, Zaibak F, Li L, Martinsson T (2005). Introduction of *in vitro* transcribed ENO1 mRNA into neuroblastoma cells induces cell death. BMC Cancer.

[181] Royds JA, Parsons MA, Taylor CB, Timperley WR (1982). Enolase isoenzyme distribution in the human brain and its tumours. J Pathol.

[182] Chang G-C, Liu K-J, Hsieh C-L, Hu T-S, Charoenfuprasert S, Liu H-K (2006). Identification of alpha-enolase as an autoantigen in lung cancer: its overexpression is associated with clinical outcomes. Clinical Cancer Res.

[183] Andreasen PA, Egelund R, Petersen HH (2000). The plasminogen activation system in tumor growth, invasion, and metastasis. Cell Mol Life Sci.

[184] Bergmann S, Rohde M, Chhatwal GS, Hammerschmidt S (2001). alpha-Enolase of Streptococcus pneumoniae is a plasmin(ogen)-binding protein displayed on the bacterial cell surface. Mol Microbiol.

[185] Pancholi V, Fischetti VA (1998). alpha-enolase, a novel strong plasmin(ogen) binding protein on the surface of pathogenic streptococci. J Biol Chem.

[186] Arza B, Félez J, Lopez-Alemany R, Miles LA, Muñoz-Cánoves P (1997). Identification of an epitope of alpha-enolase (a candidate plasminogen receptor) by phage display. Thromb Haemost.

[187] Miles LA, Dahlberg CM, Plescia J, Felez J, Kato K, Plow EF (1991). Role of cell-surface lysines in plasminogen binding to cells: identification of alpha-enolase as a candidate plasminogen receptor. Biochem.

[188] Redlitz A, Fowler BJ, Plow EF, Miles LA (1995). The role of an enolase-related molecule in plasminogen binding to cells. Europ J Biochem/FEBS.

[189] Wygrecka M, Marsh LM, Morty RE, Henneke I, Guenther A, Lohmeyer J (2009). Enolase-1 promotes plasminogen-mediated recruitment of monocytes to the acutely inflamed lung. Blood.

[190] Liu K, Shih N (2007). The Role of Enolase in Tissue Invasion and Metastasis of Pathogens and Tumor Cells. J Cancer Mol.

[191] Feo S, Arcuri D, Piddini E, Passantino R, Giallongo A (2000). ENO1 gene product binds to the c-myc promoter and acts as a transcriptional repressor: relationship with Myc promoter-binding protein 1 (MBP-1). FEBS letters.

[192] Ray R, Miller DM (1991). Cloning and characterization of a human c-myc promoter-binding protein. Mol Cell Biol.

[193] Chaudhary D, Miller DM (1995). The c-myc promoter binding protein (MBP-1) and TBP bind simultaneously in the minor groove of the c-myc P2 promoter. Biochem.

[194] Subramanian A, Miller DM (2000). Structural analysis of alpha-enolase. Mapping the functional domains involved in down-regulation of the c-myc protooncogene. J Biol Chem.

[195] Sedoris KC, Thomas SD, Miller DM (2010). Hypoxia induces differential translation of enolase/MBP-1. BMC Cancer.

[196] Ferber EC, Peck B, Delpuech O, Bell GP, East P, Schulze A (2012). FOXO3a regulates reactive oxygen metabolism by inhibiting mitochondrial gene expression. Cell Death Different.

[197] Zhang H, Gao P, Fukuda R, Kumar G, Krishnamachary B, Zeller KI (2007). HIF-1 Inhibits Mitochondrial Biogenesis and Cellular Respiration in VHL-Deficient Renal Cell Carcinoma by Repression of C-MYC Activity. Cancer Cell.

[198] Kariko K, Malkowicz S, Li W, Kuo A, Barnathan E (1993). Invasive neoplastic uroepithelial cells express high-levels of urokinase receptor and plasminogen receptor, alpha-enolase. Internat J Oncol.

[199] Tanaka M, Sugisaki K, Nakashima K (1985). Purification, characterization, and distribution of enolase isozymes in chicken. J. Biochem.

[200] Imamura K, Tanaka T (1972). Multimolecular forms of pyruvate kinase from rat and other mammalian tissues. I. Electrophoretic studies. J Biochem.

[201] Weber G (1969). Regulation of pyruvate kinase. Advances Enzyme Regulat.

[202] Prasannan CB, Villar MT, Artigues A, Fenton AW (2013). Identification of regions of rabbit muscle pyruvate kinase important for allosteric regulation by phenylalanine, detected by H/D exchange mass spectrometry. Biochemistry.

[203] Assimacopoulos-Jeannet F, Jeanrenaud B (1990). Insulin activates 6-phosphofructo-2-kinase and pyruvate kinase in the liver. Indirect evidence for an action via a phosphatase. J Biol Chem.

[204] Blair JB, Cimbala MA, Foster JL, Morgan RA (1976). Hepatic pyruvate kinase. Regulation by glucagon, cyclic adenosine 3′-5′-monophosphate, and insulin in the perfused rat liver. J Biol Chem.

[205] Irving MG, Williams JF (1973). Kinetic studies on the regulation of rabbit liver pyruvate kinase. Biochem J.

[206] Bailey E, Stirpe F, Taylor CB (1968). Regulation of rat liver pyruvate kinase. The effect of preincubation, pH, copper ions, fructose 1,6-diphosphate and dietary changes on enzyme activity. Biochem J.

[207] Brown CE, Taylor JM, Chan LM (1985). The effect of pH on the interaction of substrates and effector to yeast and rabbit muscle pyruvate kinase. Bioch Biophys Acta.

[208] Rozengurt E, Jiménez de Asúa L, Carminatti H (1969). Some kinetic properties of liver pyruvate kinase (type L). II. Effect of pH on its allosteric behavior. J Biol Chem.

[209] Kinderlerer J, Ainsworth S, Morris CN, Rhodes N (1986). The regulatory properties of yeast pyruvate kinase. Effect of pH. Biochem J.

[210] Hoshino A, Hirst JA, Fujii H (2007). Regulation of cell proliferation by interleukin-3-induced nuclear translocation of pyruvate kinase. J Biol Chem.

[211] Steták A, Veress R, Ovádi J, Csermely P, Kéri G, Ullrich A (2007). Nuclear translocation of the tumor marker pyruvate kinase M2 induces programmed cell death. Cancer Res.

[212] Chandra D, Bratton SB, Person MD, Tian Y, Martin AG, Ayres M (2006). Intracellular Nucleotides Act as Critical Prosurvival Factors by Binding to Cytochrome C and Inhibiting Apoptosome. Cell.

[213] Elmore S (2007). Apoptosis: a review of programmed cell death. Toxicologic Pathol.

[214] Kass GE, Eriksson JE, Weis M, Orrenius S, Chow SC (1996). Chromatin condensation during apoptosis requires ATP. Biochemical J.

[215] Christofk HR, Vander Heiden MG, Harris MH, Ramanathan A, Gerszten RE, Wei R (2008). The M2 splice isoform of pyruvate kinase is important for cancer metabolism and tumour growth. Nature.

[216] Christofk HR, Vander Heiden MG, Wu N, Asara JM, Cantley LC (2008). Pyruvate kinase M2 is a phosphotyrosine-binding protein. Nature.

[217] Hitosugi T, Kang S, Vander Heiden MG, Chung T-W, Elf S, Lythgoe K (2009). Tyrosine phosphorylation inhibits PKM2 to promote the Warburg effect and tumor growth. Science Signaling.

[218] Shimada N, Shinagawa T, Ishii S (2008). Modulation of M2-type pyruvate kinase activity by the cytoplasmic PML tumor suppressor protein. Genes cells.

[219] Barger JF, Plas DR (2010). Balancing biosynthesis and bioenergetics: metabolic programs in oncogenesis. Endocrine-related Cancer.

[220] Clower C V, Chatterjee D, Wang Z, Cantley LC, Vander Heiden MG, Krainer AR (2010). The alternative splicing repressors hnRNP A1/A2 and PTB influence pyruvate kinase isoform expression and cell metabolism. PNAS USA.

[221] Anastasiou D, Yu Y, Israelsen WJ, Jiang J-K, Boxer MB, Hong BS (2012). Pyruvate kinase M2 activators promote tetramer formation and suppress tumorigenesis. Nat Chem Biol.

[222] Kato H, Fukuda T, Parkison C, McPhie P, Cheng SY (1989). Cytosolic thyroid hormone-binding protein is a monomer of pyruvate kinase. PNAS USA.

[223] Lloyd MC, Alfarouk KO, Verduzco D, Bui MM, Gillies RJ, Ibrahim ME (2014). Vascular measurements correlate with estrogen receptor status. BMC cancer.

[224] David CJ, Chen M, Assanah M, Canoll P, Manley JL (2010). HnRNP proteins controlled by c-Myc deregulate pyruvate kinase mRNA splicing in cancer. Nature.

[225] Sun Q, Chen X, Ma J, Peng H, Wang F, Zha X (2011). Mammalian target of rapamycin up-regulation of pyruvate kinase isoenzyme type M2 is critical for aerobic glycolysis and tumor growth. PNAS USA.

[226] Ye J, Mancuso A, Tong X, Ward PS, Fan J, Rabinowitz JD (2012). Pyruvate kinase M2 promotes de novo serine synthesis to sustain mTORC1 activity and cell proliferation. PNAS USA.

[227] Kim JW, Tchernyshyov I, Semenza GL, Dang C V (2006). HIF-1-mediated expression of pyruvate dehydrogenase kinase: A metabolic switch required for cellular adaptation to hypoxia. Cell Metab.

[228] Lu C-W, Lin S-C, Chen K-F, Lai Y-Y, Tsai S-J (2008). Induction of pyruvate dehydrogenase kinase-3 by hypoxia-inducible factor-1 promotes metabolic switch and drug resistance. J Biol Chem.

[229] Kaelin WG, Ratcliffe PJ (2008). Oxygen Sensing by Metazoans: The Central Role of the HIF Hydroxylase Pathway. Mol Cell.

[230] Schofield CJ, Ratcliffe PJ (2004). Oxygen sensing by HIF hydroxylases. Nature Revs Mol Cell Biol.

[231] Sufan RI, Moriyama EH, Mariampillai A, Roche O, Evans AJ, Alajez NM (2009). Oxygen-independent degradation of HIF-alpha via bioengineered VHL tumour suppressor complex. EMBO Mol Med.

[232] Wilhelm G, Schulz J, Hofmann E (1971). pH-dependence of aerobic glycolysis in ehrlich ascites tumour cells. FEBS letters.

[233] López-Lázaro M (2010). A new view of carcinogenesis and an alternative approach to cancer therapy. Molecular Med (Cambridge, Mass).

[234] Che X-F, Zheng C-L, Akiyama S-I, Tomoda A (2011). 2-Aminophenoxazine-3-one and 2-amino-4,4α-dihydro-4α,7-dimethyl-3H-phenoxazine-3-one cause cellular apoptosis by reducing higher intracellular pH in cancer cells. Proc Japan Acad Series B, Physic Biol Sci.

[235] Warburg O (1956). On the origin of cancer cells. Science.

[236] Cori CF, Cori GT (1925). The Carbohydrate Metabolism of Tumors. I. The free sugar, lactic acid and glycogen content of malignant tumors. J Biol Chem.

[237] Goldfeder A (1933). Theoretical basis for the acidotic treatment of neoplasia. Am J Surg.

[238] Burk D. A (1939). A colloquial consideration of the Pasteur and neo-Pasteur effects. Cold Spring Harbor Symposia Quantit Biol.

[239] Eagle H (1973). The effect of environmental pH on the growth of normal and malignant cells. J Cell Physiol.

[240] Eagle H (1974). Some effects of environmental pH on cellular metabolism and function. Control of Proliferation in Animal Cells. Control of Proliferation in Animal Cells.

[241] Gatenby RA, Gillies RJ (2004). Why do cancers have high aerobic glycolysis?. Nature Revs Cancer.

[242] Calderon-Montano JM, Burgos-Moron E, Perez-Guerrero C, Salvador J, Robles A, Lopez-Lazaro M (2011). Role of the Intracellular pH in the Metabolic Switch between Oxidative Phosphorylation and Aerobic Glycolysis - Relevance to Cancer. WebmedCentral CANCER.

[243] Warburg O (1931). Nobel Lecture.

[244] Iessi E, Marino ML, Lozupone F, Fais S (2008). Tumor acidity and malignancy : novel aspects in the design of anti-tumor therapy Review Article. Cancer Ther.

[245] Marino ML, Pellegrini P, Di Lernia G, Djavaheri-Mergny M, Brnjic S, Zhang X (2012). Autophagy is a protective mechanism for human melanoma cells under acidic stress. J Biol Chem.

[246] De Milito A, Canese R, Marino ML, Borghi M, Iero M, Villa A (2010). pH-dependent antitumor activity of proton pump inhibitors against human melanoma is mediated by inhibition of tumor acidity. Internat J Cancer.

[247] Xu K, Mao X, Mehta M, Cui J, Zhang C, Mao F (2013). Elucidation of how cancer cells avoid acidosis through comparative transcriptomic data analysis. PLOS One.

[248] Ristow M (2006). Oxidative metabolism in cancer growth. Curr Opinion Clinic Nutrition Metabolic Care.

[249] Moreno-Sánchez R, Rodríguez-Enríquez S, Marín-Hernández A, Saavedra E (2007). Energy metabolism in tumor cells. The FEBS journal.

[250] Hsu PP, Sabatini DM (2008). Cancer cell metabolism: Warburg and beyond. Cell.

[251] Kritikou E (2008). Metabolism: Warburg effect revisited. Nature Revs Cancer.

[252] Kroemer G, Pouyssegur J (2008). Tumor Cell Metabolism: Cancer's Achilles' Heel. Cancer Cell.

[253] Tennant DA, Durán R V, Gottlieb E (2010). Targeting metabolic transformation for cancer therapy. Nature Revs Cancer.

[254] Koppenol WH, Bounds PL, Dang C V (2011). Otto Warburg's contributions to current concepts of cancer metabolism. Nature Revs Cancer.

[255] Harguindey S, Orive G, Luis Pedraz J, Paradiso A, Reshkin SJ (2005). The role of pH dynamics and the Na+/H+ antiporter in the etiopathogenesis and treatment of cancer. Two faces of the same coin--one single nature. Biochim Biophys Acta.

[256] Parks SK, Chiche J, Pouyssegur J (2011). pH control mechanisms of tumor survival and growth. J Cell Physiol.

[257] Izumi H, Torigoe T, Ishiguchi H, Uramoto H, Yoshida Y, Tanabe M (2003). Cellular pH regulators: potentially promising molecular targets for cancer chemotherapy. Cancer Treat Revs.

[258] Parks SK, Chiche J, Pouysségur J (2013). Disrupting proton dynamics and energy metabolism for cancer therapy. Nature Revs Cancer.

[259] Moriyama Y, Nelson N (1988). Inhibition of vacuolar H+-ATPases by fusidic acid and suramin. FEBS letters.

[260] Pedersen SF, Stock C (2013). Ion channels and transporters in cancer: pathophysiology, regulation, and clinical potential. Cancer Res.

[261] Asaumi J, Kawasaki S, Nishikawa K, Kuroda M, Hiraki Y (1995). Influence of the extracellular pH, an inhibitor of Na+/H+ exchanger and an inhibitor of Cl-/HCO3-exchanger on adriamycin accumulation. Anticancer Res.

[262] Stock C, Cardone RA, Busco G, Krähling H, Schwab A, Reshkin SJ (2008). Protons extruded by NHE1: digestive or glue?. European J Cell Biol.

[263] Stock C, Ludwig FT, Schwab A (2012). Is the multifunctional Na(+)/H(+) exchanger isoform 1 a potential therapeutic target in cancer?. Current Med Chem.

[264] Boedtkjer E, Bunch L, Pedersen SF (2012). Physiology, pharmacology and pathophysiology of the pH regulatory transport proteins NHE1 and NBCn1: similarities, differences, and implications for cancer therapy. Current Pharm Design.

[265] Harguindey S, Cragoe EJ (1992). The Na+/H+ antiporter in oncology in the light of the spontaneous regression of cancer and cell metabolism. Med Hypoth.

[266] McLean LA, Roscoe J, Jorgensen NK, Gorin FA, Cala PM (2000). Malignant gliomas display altered pH regulation by NHE1 compared with nontransformed astrocytes. American journal of physiology Cell Physiol.

[267] Rauch C, Blanchard A, Wood E, Dillon E, Wahl ML, Harguindey S, Meszaros Agoston, Balogh Gusztav (2009). Cell Membranes, Cytosolic pH and Drug Transport in Cancer and MDR: Physics, Biochemistry and Molecular Biology. Multiple Drug Resistance.

[268] Amith SR, Fliegel L (2013). Regulation of the Na+/H+ Exchanger (NHE1) in Breast Cancer Mets. Cancer Res.

[269] Daniel C, Bell C, Burton C, Harguindey S, Reshkin SJ, Rauch C (2013). The role of proton dynamics in the development and maintenance of multidrug resistance in cancer. Biochim Biophys Acta.

[270] Doppler W, Jaggi R, Groner B (1987). Induction of v-mos and activated Ha-ras oncogene expression in quiescent NIH 3T3 cells causes intracellular alkalinisation and cell-cycle progression. Gene.

[271] Hagag N, Lacal JC, Graber M, Aaronson S, Viola M V (1987). Microinjection of ras p21 induces a rapid rise in intracellular pH. Mol Cell Biol.

[272] Orive G, Reshkin SJ, Harguindey S, Pedraz JL (2003). Hydrogen ion dynamics and the Na+/H+ exchanger in cancer angiogenesis and antiangiogenesis. British J Cancer.

[273] Mo X-G, Chen Q-W, Li X-S, Zheng M-M, Ke D-Z, Deng W (2011). Suppression of NHE1 by small interfering RNA inhibits HIF-1α-induced angiogenesis *in vitro* via modulation of calpain activity. Microvascular Res.

[274] Rich IN, Worthington-White D, Garden OA, Musk P (2000). Apoptosis of leukemic cells accompanies reduction in intracellular pH after targeted inhibition of the Na(+)/H(+) exchanger. Blood.

[275] Harguindey S, Orive G, Cacabelos R, Hevia EM, de Otazu RD, Arranz JL (2008). An integral approach to the etiopathogenesis of human neurodegenerative diseases (HNDDs) and cancer. Possible therapeutic consequences within the frame of the trophic factor withdrawal syndrome (TFWS). Neuropsychiatric Dis Treat.

[276] Harguindey S, Cragoe E. J., Kleyman Th. R, Simchowitz L (1992). Use of Na+/H+ antiporter inhibitors as a novel approach to cancer treatment. Amiloride and Its Analogs: Unique Cation Transport Inhibitors.

[277] Provost JJ, Wallert M A (2013). Inside out: targeting NHE1 as an intracellular and extracellular regulator of cancer progression. Chemical Biol Drug Design.

[278] Loo (2012). NHE-1: A Promising Target for Novel Anti-cancer Therapeutics. Curr Pharm Des.

[279] Provost JJ, Rastedt D, Canine J, Ngyuen T, Haak A, Kutz C, Berthesen N, Slusser A, Andersen K, Dorsam G, Wallert MA (2012). Urokinase plasminogen activator receptor induced non-small cell lung cancer invasion and metastasis requires NHE1 transporter expression and transport activity. Cellular Oncol (Dordr).

[280] Jankun J, Skrzypczak-Jankun E (1999). Molecular basis of specific inhibition of urokinase plasminogen activator by amiloride. Cancer Biochem Biophys.

[281] Kim T-D, Song K-S, Li G, Choi H, Park H-D, Lim K (2006). Activity and expression of urokinase-type plasminogen activator and matrix metalloproteinases in human colorectal cancer. BMC Cancer.

[282] He B, Deng C, Zhang M, Zou D, Xu M (2007). Reduction of intracellular pH inhibits the expression of VEGF in K562 cells after targeted inhibition of the Na+/H+ exchanger. Leukemia Res.

[283] Kellen JA, Mirakian A, Kolin A Antimetastatic effect of amiloride in an animal tumour model. Anticancer Res.

[284] Harguindey S, Orive G, Pedraz J, Bello G, Arranz J SJ (2002). Apparent cure of a case of metastatic ovarian carcinoma after chronic treatment with Na+–H+ antiporter inhibitors. Oncologia (Madrid).

[285] Matthews H, Ranson M, Kelso MJ (2011). Anti-tumour/metastasis effects of the potassium-sparing diuretic amiloride: an orally active anti-cancer drug waiting for its call-of-duty?. Internat J Cancer.

[286] Alliegro MC, Alliegro MA, Cragoe EJ, Glaser BM (1993). Amiloride inhibition of angiogenesis *in vitro*. J Experiment Zool.

[287] Junior J (2010). Metastatic neuroendocrine carcinoma of the pancreas - case report and literature review. Rev Brasil Oncol.

[288] Bellizzi A, Mangia A, Malfettone A, Cardone RA, Simone G, Reshkin SJ, Paradiso A (2011). Na+/H+ exchanger regulatory factor 1 expression levels in blood and tissue predict breast tumour clinical behaviour. Histopathol.

[289] Tomoda A, Miyazawa K, Tabuchi T (2013). Prevention of carcinogenesis and development of gastric and colon cancers by 2-aminophenoxazine-3-one (Phx-3): direct and indirect anti-cancer activity of Phx-3. Internat J Mol Sci.

[290] Atwal KS, O'Neil S V, Ahmad S, Doweyko L, Kirby M, Dorso CR (2006). Synthesis and biological activity of 5-aryl-4-(4-(5-methyl-1H-imidazol-4-yl)piperidin-1-yl)pyrimidine analogs as potent, highly selective, and orally bioavailable NHE-1 inhibitors. Bioorg Med Chem Letts.

[291] Harguindey S, Polo Orozco JD, Macías FA, Gonzalez-Molinillo JM, Chinchilla D, Reshkin SR, Tomoda A (2011). Further along a clinical protocol using a cocktail of PTIs (Proton Transport Inhibitors) in human cancer.

[292] Porporato PE, Dhup S, Dadhich RK, Copetti T, Sonveaux P (2011). Anticancer targets in the glycolytic metabolism of tumors: a comprehensive review. Frontiers Pharmacol.

[293] Busco G, Cardone RA, Greco MR, Bellizzi A, Colella M, Antelmi E, Mancini MT, Dell'Aquila ME, Casavola V, Paradiso A, Reshkin SJ (2010). NHE1 promotes invadopodial ECM proteolysis through acidification of the peri-invadopodial space. The FASEB J.

[294] Lucien F, Brochu-Gaudreau K, Arsenault D, Harper K, Dubois CM (2011). Hypoxia-induced invadopodia formation involves activation of NHE-1 by the p90 ribosomal s6 kinase (p90RSK). PLoS One.

[295] Jin W, Li Q, Wang J, Chang G, Lin Y, Li H (2012). Na+/H+ exchanger 1 inhibition contributes to K562 leukaemic cell differentiation. Cell Biol Intern.

[296] Di Sario A, Bendia E, Omenetti A, De Minicis S, Marzioni M, Kleemann HW, Candelaresi C, Saccomanno S, Alpini G, Benedetti A (2007). Selective inhibition of ion transport mechanisms regulating intracellular pH reduces proliferation and induces apoptosis in cholangiocarcinoma cells. Digestive Liver Dis.

[297] Zeymer U, Suryapranata H, Monassier JP, Opolski G, Davies J, Rasmanis G, Linssen G, Tebbe U, Schröder R, Tiemann R, Machnig T, Neuhaus KL (2001). The Na(+)/H(+) exchange inhibitor eniporide as an adjunct to early reperfusion therapy for acute myocardial infarction. Results of the evaluation of the safety and cardioprotective effects of eniporide in acute myocardial infarction (ESCAMI) trial. J American College Cardiol.

[298] Rupprecht HJ, vom Dahl J, Terres W, Seyfarth KM, Richardt G, Schultheibeta HP, Buerke M, Sheehan FH, Drexler H (2000). Cardioprotective effects of the Na(+)/H(+) exchange inhibitor cariporide in patients with acute anterior myocardial infarction undergoing direct PTCA. Circulat.

[299] Boyce SW, Bartels C, Bolli R, Chaitman B, Chen JC, Chi E, Jessel A, Kereiakes D, Knight J, Thulin L, Theroux P (2003). Impact of sodium-hydrogen exchange inhibition by cariporide on death or myocardial infarction in high-risk CABG surgery patients: Results of the CABG surgery cohort of the GUARDIAN study. J Thoracic Cardiovasc Surg.

[300] Chaitman BR A review of the GUARDIAN trial results: clinical implications and the significance of elevated perioperative CK-MB on 6-month survival. J Cardiac Surg.

[301] Mentzer RM, Bartels C, Bolli R, Boyce S, Buckberg GD, Chaitman B, Haverich A, Knight J, Menasché P, Myers ML, Nicolau J, Simoons M, Thulin L (2008). Sodium-Hydrogen Exchange Inhibition by Cariporide to Reduce the Risk of Ischemic Cardiac Events in Patients Undergoing Coronary Artery Bypass Grafting: Results of the EXPEDITION Study. Ann Thoracic Surg.

[302] Reshkin SJ, Bellizzi A, Cardone RA, Tommasino M, Casavola V, Paradiso A (2003). Paclitaxel induces apoptosis via protein kinase A- and p38 mitogen-activated protein-dependent inhibition of the Na+/H+ exchanger (NHE) NHE isoform 1 in human breast cancer cells. Clinical Cancer Res.

[303] Théroux P, Chaitman BR, Danchin N, Erhardt L, Meinertz T, Schroeder JS, Tognoni G, White HD, Willerson JT, Jessel A (2000). Inhibition of the sodium-hydrogen exchanger with cariporide to prevent myocardial infarction in high-risk ischemic situations. Main results of the GUARDIAN trial. Guard during ischemia against necrosis (GUARDIAN) Investigators. Circulat.

[304] Dhein S, Salameh A (1999). Na + / H + -Exchange Inhibition by Cariporide (Hoe 642): A New Principle in Cardiovascular Medicine. Cardiovasc Drug Revs.

[305] Avkiran M, Cook AR, Cuello F (2008). Targeting Na+/H+ exchanger regulation for cardiac protection: a RSKy approach?. Current Opinion Pharmacol.

[306] Humphreys RA, Haist J V, Chakrabarti S, Feng Q, Arnold JM, Karmazyn M (1999). Orally administered NHE1 inhibitor cariporide reduces acute responses to coronary occlusion and reperfusion. American J Physiol.

[307] Baartscheer A, Schumacher CA, van Borren MMGJ, Belterman CNW, Coronel R, Opthof T, Fiolet JWT (2005). Chronic inhibition of Na+/H+-exchanger attenuates cardiac hypertrophy and prevents cellular remodeling in heart failure. Cardiovasc Res.

[308] Kilic A, Velic A, De Windt LJ, Fabritz L, Voss M, Mitko D (2005). Enhanced activity of the myocardial Na+/H+ exchanger NHE-1 contributes to cardiac remodeling in atrial natriuretic peptide receptor-deficient mice. Circulation.

[309] Lv C, Yang X, Yu B, Ma Q, Liu B, Liu Y (2012). Blocking the Na+/H+ exchanger 1 with cariporide (HOE642) reduces the hypoxia-induced invasion of human tongue squamous cell carcinoma. Internat J Oral Maxillofacial Surg.

[310] Shi Q, Le X, Wang B, Abbruzzese JL, Xiong Q, He Y (2001). Regulation of vascular endothelial growth factor expression by acidosis in human cancer cells. Oncogene.

[311] Xu L, Fukumura D, Jain RK (2002). Acidic extracellular pH induces vascular endothelial growth factor (VEGF) in human glioblastoma cells via ERK1/2 MAPK signaling pathway: mechanism of low pH-induced VEGF. J Biol Chem.

[312] Yang X, Wang D, Dong W, Song Z, Dou K (2010). Inhibition of Na(+)/H(+) exchanger 1 by 5-(N-ethyl-N-isopropyl) amiloride reduces hypoxia-induced hepatocellular carcinoma invasion and motility. Cancer Letts.

[313] Spugnini EP, Sonveaux P, Stock C, Perez-Sayans M, De Milito A, Avnet S, Garcìa AG, Harguindey S, Fais S (2014). Proton channels and exchangers in cancer. Biochim Biophys Acta (BBA) Biomembranes.

[314] Pouysségur J, Dayan F, Mazure NM (2006). Hypoxia signalling in cancer and approaches to enforce tumour regression. Nature.

[315] Benjamin DJ (2014). The efficacy of surgical treatment of cancer - 20 years later. Medical Hypotheses.

[316] Gatenby RA (2009). A change of strategy in the war on cancer. Nature.

[317] Harguindey S (1994). Unitarian strategy of cancer cells; carbohydrate metabolism: (first of three parts). Ann Int Med (Madrid).

[318] Fais S, Venturi G, Gatenby R (2014). Microenvironmental acidosis in carcinogenesis and metastases: new strategies in prevention and therapy. Cancer Metast Rev.

[319] Otto AM, Hintermair J, Janzon C (2014). NADH-Linked Metabolic Plasticity of MCF-7 Breast Cancer Cells Surviving in a Nutrient-Deprived Microenvironment. J Cell Biochem.

