# Glycolysis, tumor metabolism, cancer growth and dissemination. A new pH-based etiopathogenic perspective and therapeutic approach to an old cancer question

**DOI:** 10.18632/oncoscience.158

**Published:** 2014-12-18

**Authors:** Khalid O. Alfarouk, Daniel Verduzco, Cyril Rauch, Abdel Khalig Muddathir, Adil H. H. Bashir, Gamal O. Elhassan, Muntaser E. Ibrahim, Julian David Polo Orozco, Rosa Angela Cardone, Stephan J. Reshkin, Salvador Harguindey

Erratum: Glycolysis, tumor metabolism, cancer growth and dissemination. A new pH-based etiopathogenic perspective and therapeutic approach to an old cancer question

Khalid O. Alfarouk, Daniel Verduzco, Cyril Rauch, Abdel Khalig Muddathir, Adil H. H. Bashir, Gamal O. Elhassan, Muntaser E. Ibrahim, Julian David Polo Orozco, Rosa Angela Cardone, Stephan J. Reshkin, and Salvador Harguindey

Oncoscience. 2014; 1(12): 777–802.

PMCID: PMC4303887 PMID: 25621294

In Figure 5 the depicted molecules do not correspond to the legend.

The corrected Figure is provided here.

**Figure 5:**
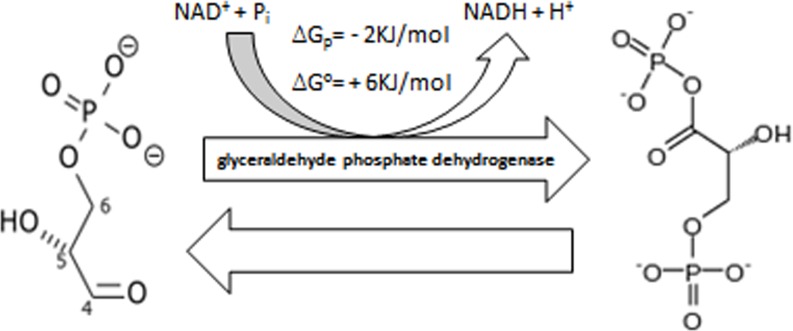
Conversion of D-glyceraldehyde 3-phosphate (GADP) into into 1, 3-bisphosphoglycerate

